# Rational design and synthesis of a novel BODIPY-based probe for selective imaging of tau tangles in human iPSC-derived cortical neurons

**DOI:** 10.1038/s41598-022-09016-z

**Published:** 2022-03-28

**Authors:** Alessandro Soloperto, Deborah Quaglio, Paola Baiocco, Isabella Romeo, Mattia Mori, Matteo Ardini, Caterina Presutti, Ida Sannino, Silvia Ghirga, Antonia Iazzetti, Rodolfo Ippoliti, Giancarlo Ruocco, Bruno Botta, Francesca Ghirga, Silvia Di Angelantonio, Alberto Boffi

**Affiliations:** 1 Center for Life Nano- & Neuro-Science, Istituto Italiano Di Tecnologia, 00161 Rome, Italy; 2 Department of Chemistry and Technology of Drugs, Department of Excellence 2018–2022, Sapienza University of Rome, 00185 Rome, Italy; 3 Department of Biochemical Sciences “A. Rossi Fanelli”, Sapienza University of Rome, 00185 Rome, Italy; 4 Department of Biotechnology, Chemistry and Pharmacy, Department of Excellence 2018–2022, University of Siena, 53100 Siena, Italy; 5 Department of Life, Health, and Environmental Sciences, University of L’Aquila, 67100 L’Aquila, Italy; 6 Department of Basic Biotechnological Sciences, Intensivological and Perioperative Clinics, Catholic University of Sacred Heart, 00168 Rome, Italy; 7 Department of Physiology and Pharmacology, Sapienza University of Rome, 00185 Rome, Italy

**Keywords:** Cellular neuroscience, Diseases of the nervous system, Stem cells in the nervous system, Computational chemistry, Protein folding, Small molecules, Biosynthesis, Chemical synthesis

## Abstract

Numerous studies have shown a strong correlation between the number of neurofibrillary tangles of the tau protein and Alzheimer's disease progression, making the quantitative detection of tau very promising from a clinical point of view. However, the lack of highly reliable fluorescent probes for selective imaging of tau neurofibrillary tangles is a major challenge due to sharing similar β–sheet motifs with homologous Amyloid-β fibrils. In the current work, we describe the rational design and the in silico evaluation of a small-size focused library of fluorescent probes, consisting of a BODIPY core (electron acceptor) featuring highly conjugated systems (electron donor) with a length in the range 13–19 Å at C3. Among the most promising probes in terms of binding mode, theoretical affinity and polarity, BT1 has been synthesized and tested in vitro onto human induced pluripotent stem cells derived neuronal cell cultures. The probe showed excellent photophysical properties and high selectivity allowing in vitro imaging of hyperphosphorylated tau protein filaments with minimal background noise. Our findings offer new insight into the structure-activity relationship of this class of tau selective fluorophores, paving the way for boosting tau tangle detection in patients possibly through retinal spectral scans.

## Introduction

Alzheimer's disease (AD) is a neurodegenerative disorder responsible for more than 80% of cases of senile dementia. Despite the presence of extracellular β-amyloid (Aβ) and intracellular tau protein aggregates is an established hall-mark of the AD pathology, the limited scientific evidence on the timing of the molecular cascade leading to AD-related neurodegeneration has made difficult the development of both early diagnostic strategies and effective therapies.

To date, no solutions have been found that clearly hinders disease progression thus improving the life quality of patients and caregivers. Moreover, most of the drugs developed in rodent AD models failed in reducing disease burden in clinical trials, likely due to the lack of globally recognized diagnostic criteria in pre-symptomatic phases of AD^1,2^. Indeed, the only drug recently approved by FDA, Aducanumab, has been shown to reduce Aβ plaques in people showing early Alzheimer’s disease together with evident buildup of Aβ plaques. Moreover, Aducanumab, has been approved, despite a number of side effects including amyloid-related imaging abnormalities, headache, dizziness, nausea, confusion and vision changes.

Consequently, the current challenge is the development of non-invasive *in vivo* approaches that can ensure the assessment of small, still undetectable, neuropathological changes in living patients. Indeed, to date, a definitive diagnosis for AD can be made only post-mortem, by immunohistochemical analysis of brain samples^3^.

Thus, numerous efforts are now exploiting cerebrospinal fluid analysis (CSF)^4^ and cutting-edge diagnostic neuroimaging techniques^5^, for the detection of the presence of Aβ and/or tau protein aggregates in living AD patients. However, despite being relatively specific and selective, these approaches display several limitations including high costs of management and invasiveness. In 2018, the National Institute on Aging and Alzheimer’s Association confirmed a biological definition of AD and established a consensus on bioprobe-based diagnostic criteria for interventional research^6^. Three main biomarkers have been identified for AD, namely: (i) the Aβ42 peptide, which is the main component of the toxic Aβ plaques; (ii) the coupled units of hyperphosphorylated tau protein (P-tau) and total-tau monomers (T-tau) which are the key bricks of the neurofibrillary tangles (NFTs).

The amyloid cascade hypothesis, the Aβ hypothesis, has been the mainstream explanation for the AD pathogenesis for over 25 years^7^; however, except for Aducanumab, all attempts to develop Aβ-targeting drugs to treat AD failed in clinical trials, thus suggesting that Aβ cannot be considered as main the factor underlying the development and progression of AD. It has to be considered that the Aβ hypothesis, involving intracellular β and γ secretases, is consistent with familial AD cases, where pathogenetic mutations of APP are clustered near the cleavage sites of secretases, and are associated with an increase in the ratio of Aβ42 formation. Interestingly, Down’s syndrome patients with trisomy 21 exhibit AD-like pathology by about 40 years of age^8^. In addition, other familial AD mutations, identified in presenilin 1/2, which is a component of γ-secretase, are closely linked to the Aβ production process, providing a rational basis for the idea that Aβ amyloid fibril formation can account for the familiar AD pathogenesis^7^. However, although transgenic mice expressing familial AD mutations on these genes display accumulation of Aβ plaques, intracellular tau tangles, and neuronal death have not been observed in their brains^9^. Indeed, the only AD mouse model characterized by tau pathology is a triple transgenic mouse expressing specifically in the MAPT gene the P301L mutation, which, in patients, relates to familial frontotemporal dementia (3xTg-AD mouse)^10,11^. Thus, it is now accepted that extra-cellular accumulation of Aβ fibrils does not induce tau accumulation; consistently, antibody-based pharmacological treatments targeting Aβ in AD mice decreased brain Aβ deposition, leaving unaltered tau accumulation^12–15^. Strikingly, the detection of Aβ amyloid accumulation in the living patient’s brain with recent imaging tools, showed AD patients with little amyloid deposits, while elderly non-demented patients with a distribution of senile plaques as extensive as that of dementia patients^16–18^. Based on this evidence, the amyloid hypothesis of neurodegeneration/neuronal loss and amyloid deposition appear to be independent phenomena^18^.

On the other hand, numerous studies have shown a more rigorous correlation between the number of neurofibrillary tangles of the tau protein and disease progression, making the quantitative detection of tau very promising from a clinical point of view^19^. Moreover, this could be useful for disease prediction and staging as well as for the evaluation of the effectiveness of medical treatments. Therefore, tau visualization on a temporal and spatial scale has the potential to become a prognostic tool. To address this issue, positron emission tomography (PET) imaging exploits isotope-conjugated tracers that bind tau molecules with high affinity and specificity. However, the structural organization and the intracellular localization of tau protein constitute a huge challenge for the development of reliable radio-tracers^20,21^.

Over the last decade, significant progress has been made in the field of small molecule fluorescent sensors for targeting aggregated tau^22,23^.

Different chemical strategies have been used to develop affordable probes/methodologies that could be potentially used for the diagnosis of tau-based neurodegenerative disease^22,24,25^. Among them, near-infrared probes (NIR), characterized by emission wavelengths in the 650–900 nm range, seem to be good candidate probes for *in vivo* application. Indeed, emission wavelengths falling in this range facilitate tissue penetration, reducing autofluorescence and light scattering^26^. Curcumin, a natural pigment derived from the rizoma Curcuma longa, has been reported to label Aβ and tau protein aggregates. Specifically, a NIR tracer for tau aggregates has been proposed based on structural modification of CRANAD-2, a curcumin-based probe selective for beta-amyloid^27^. A number of derivatives, characterized by a significant change in fluorescence upon binding to tau fibrils, have been proposed; specifically, derivative 1c was slightly off from the NIR range (~620 nm)^25^; 3g and 3h probes revealed good colabeling with an antibody against P-tau^28^; and the 2e compound, with high specificity for tau over beta-amyloid, displayed fluorescence emission (λem = 660 nm) and a large Stokes shift (110 nm)^29^.

The main detection strategy has been based on the development of probes intercalating into P-tau aggregates^30^. The design of NFT binders has been guided by two hypotheses: (i) probes having a distance of 13−19 Å between the donor and acceptor selectively target NFTs, while probes characterized by a shorter distance favor the detection of Aβ plaques; (ii) fused ring containing probes show higher selectivity for tau over Aβ fibrils. However, due to the intrinsically disordered nature of NFTs, a clear structure–activity relationship for tau protein has not been identified to date, making the rational design of specific fluorescence probes challenging.

Indeed, one major concern is represented by the nonspecific binding of developed ligands due to the presence of β-sheet motifs, a common feature of protein aggregates other than NFTs (such as TDP-43 and α-synuclein). Moreover, heterogeneous structural organization of tau aggregates has been observed in AD and non-AD-related tauopathies. Thus, the need for non-toxic fluorescence dyes bearing better selectivity, affinity, and specificity for tau proteins is fostering the development of a novel generation of tracers^31,32^. In this regard, the recent characterization of tau protein structure at the atomic^33,34^ might represent an outstanding milestone for better tackling such needs^35^. A novel approach consists in the development of compounds based on boron dipyrrin platform (4,4-difluoro-4-bora-3a,4a-diaza-s-indacene, also known as BODIPY) that are endowed with spectroscopic and photodynamic features that can be easily customized through relatively simple chemical reactions thus making them suitable for several applications^36^. Based on molecular docking observations, the synthesis of two BODIPY-based probes selective for tau (TAU1 and TAU2) has been described^37^. Specifically, TAU1 has been reported to label P-tau in the 3xTg-AD mouse model, opening up avenues for the cost-effective monitoring of the tau protein aggregation state in animal models and possibly in human fixed tissue staining^37^. Recently, the development of non-toxic tau tracers suitable for *in vivo* applications is also boosted by clinical evidence showing the presence of tau aggregates in postmortem samples of AD patients’ retina^38–41^. Indeed, the retinal imaging of tau aggregates in living patients could play a dramatic role in AD diagnosis^41,42^, and BODIPY based probes may represent a valuable tool for this purpose. However, chemical aspects such as binding selectivity, short emission wavelength and poor solubility must be taken into account, as they represent a clear obstacle to overcome for further human biomedical application. Concerning the use of chemicals for diagnostic and therapeutic purposes in human patients, it has to be considered that the failure of pre-clinical trials of most of the potential drug candidates, today screened on AD mouse models^43^, arises also from physiological and evolutionary species-specific differences that hamper translating to humans the results obtained in rodents. Therefore, the need to develop a humanized AD model is rapidly growing, and would aid to better understand the molecular cascade and thus accelerate the screening process for diagnostic and therapeutic candidate molecules. In this framework, human-induced Pluripotent Stem Cells (hiPSCs)^44^ have emerged, in the last decade, as useful tools for modeling human neurological disorders, including AD. Indeed, by introducing specific disease-causing genomic alterations into the hiPSC line of choice by gene editing, and differentiating iPSCs into the neuronal, glial, or retinal cells, it is possible to provide a straightforward approach for unmasking phenotypic and functional alterations characteristic of complex diseases.

We here describe the rational design of a small-size focused library of fluorescent probes, to target P-tau, consisting of a BODIPY core functionalized in position C3 with a highly conjugated system ending with an amino group, and characterized by a distance in the range 13-19 Å between the electron donor and the acceptor portion. The interaction between the aforementioned BODIPY series and the crystallographic structures^33^ of the hexapeptide fragment PHF6, present in the R3 region of the P-tau protein and responsible for the propensity of the protein itself to assemble into fibrils, has been studied by molecular docking. Such a study indicated hit compounds within the *in silico* generated collection. Among the most promising probes in terms of binding mode, theoretical affinity and polarity, BT1 has been selected as a model compound for the series and has been synthesized and tested *in vitro* onto human iPSC-derived NGN2-induced neuronal cell cultures after 30 days in culture. The probes showed excellent photophysical properties and high selectivity allowing *in vitro* imaging of hyperphosphorylated tau protein filaments with minimal background noise. This study provides new insights into the structure-activity relationship of this class of tau selective fluorophores.

## Results

### Rational design of a small-size focused library of BODIPY fluorescent probes selective for NFTs

**Figure 1:**
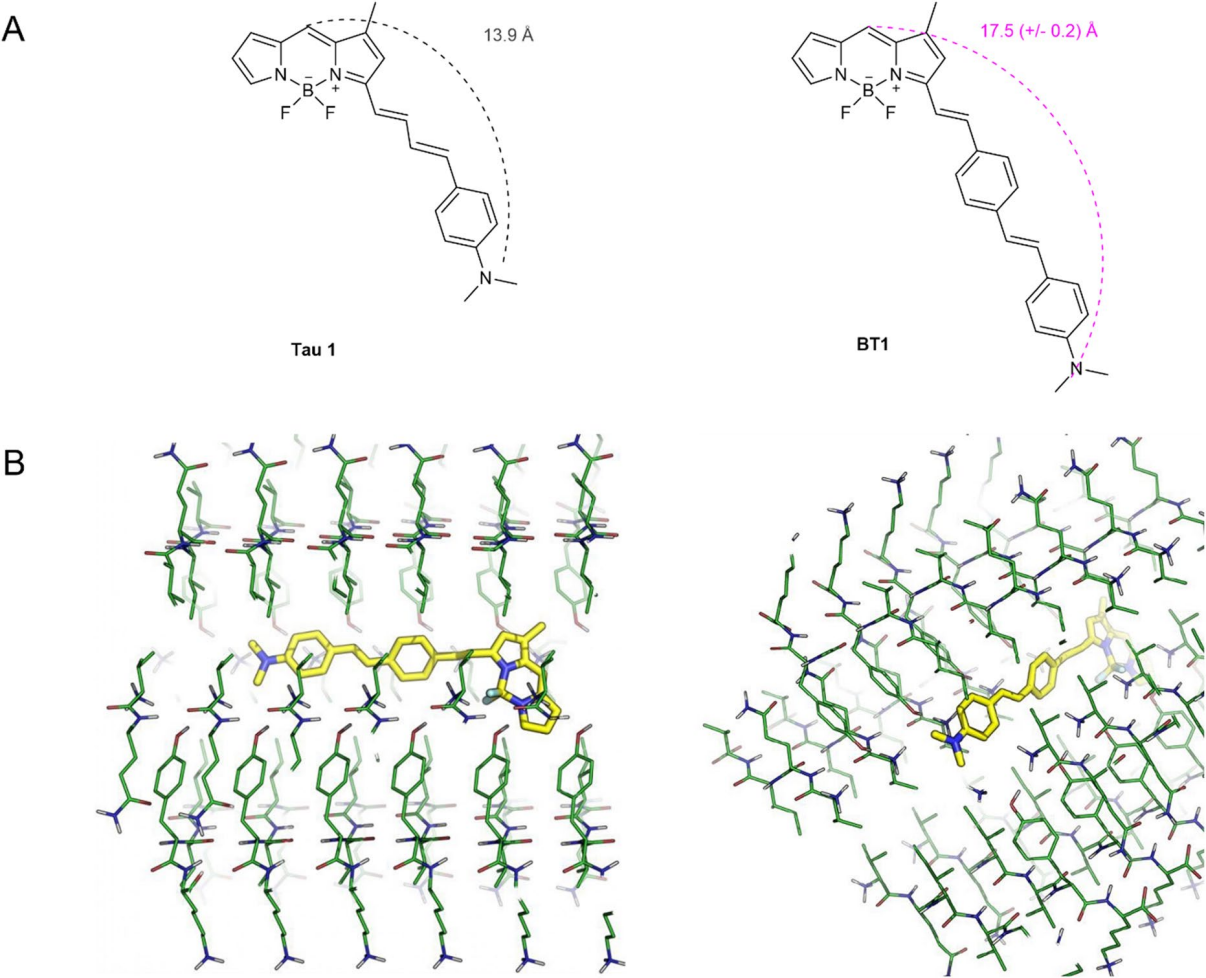
Chemical structures of BODIPY based fluorophores. (**A**) Chemical structures of TAU1 and BT1. (created by ChemDraw version 20.1.0.112 (112); PerkinElmer Inc) (**B**) Molecular docking of BT1 against the crystallographic structure of the PHF6 fragment: side and diagonal view of the molecule in the protein tunnel. (PyMOL Software. The PyMOL Molecular Graphics System, Version 2.2.0 Schrödinger, LLC. URL: https://pymol.org/2/).

Currently, a pharmacophoric model for the interaction between fluorescent probes and NFTs has not been defined and potential selective probes characterized by high chemical diversity have been identified. However, these compounds have several common structural features that allow defining some guidelines for the rational design of new probes^22^. Based on these, a small-size focused library of eight new BODIPY probes, BT1-8 (Figs. 1 and Supplementary Fig. S1), has been designed by extending the conjugation of position 3 of the BODIPY core characteristic of a known selective fluorescent probe, TAU1 (Fig. 1), in order to gain new insights into the structure-activity relationship of this fancy class of fluorophores. The series of fluorescent probes here designed, consisted of a BODIPY core functionalized in position 3 with a highly conjugated system ending with an aliphatic amine, cyclic and non, or aromatic, characterized by a distance between the electron donor portion and the acceptor portion of 13-19 Å, to improve the P-tau vs Aβ selectivity, and by a different polarity.

### Molecular docking

The detection strategy for NFTs is not as clear as it is for Aβ aggregates, probably due to the less defined binding modes in the case of P-tau aggregates. The most challenging aspect of the approach based on the intercalation of a selective probe is to ensure selective binding affinity toward P-tau aggregates over Aβ plaques. To overcome this issue, *in silico* pre-screening of the chemical library was carried out to prioritize a few compounds for chemical synthesis and biological testing, based on their predicted binding mode and theoretical affinity for the target protein. Following the work of Verwilst et al, the small library of BODIPY-based probes was docked toward the 6-mer model of the PHF6 fragment, present in the R3 region of the P-tau protein and responsible for the propensity of the protein itself to assemble into fibrils^37^. Docking was analyzed both in terms of binding mode and theoretical affinity with the aim of identifying the most promising molecule as a selective P-tau probe (Fig. 1, Supplementary Figs. S1–10 and Supplementary Table 1). All the tested molecules were able to interact with the protein system *in silico*, and to fit the cavity formed by the assembly of multiple polypeptide units. In particular, the fluorescent moiety of the compounds was mostly docked into the central part of the binding site, suggesting that it has a preference for lipophilic regions of the target protein, while the aliphatic chain was projected towards the entrance of the lipophilic tunnel (Supplementary Figs. S2–S10). The docking of the BT5, BT7, and BT8 (pose 2) compounds in their protonated form provided a weaker theoretical binding affinity than other neutral compounds (see Supporting Information, Table 1) probably due to the electrostatic repulsion between the protonated ligands’ moiety and the positively charged N-terminal ends of valine residues that are exposed within the accessible side of the protein tunnel.

BT2 provided a better theoretical binding affinity than other probes, but it presented two statistically most relevant docking poses in which the BODIPY core binds with opposite orientation near the entrance of the lipophilic tunnel. Indeed, in the second pose the aliphatic chain ending with a bulky and hydrophobic diphenylamine moiety is projected deeply in the central part of the receptor. Moreover, BT2 has significantly lower solubility in the buffers that can be used in experiments with living cells and tissues compared to BT1 (CLogP = 8.716 and 12.189 for BT1 and BT2, respectively). Accordingly, combining the predicted affinity and binding mode with the polarity, we decided to synthesize BT1 as a model compound of the series, and to investigate its selectivity towards the neurofibrillary aggregates of P-tau protein *in vitro* onto iPSC-derived NGN2-induced neuronal cell cultures after 30 days in culture.

### Synthesis and fluorescence of BT1

**Figure 2:**
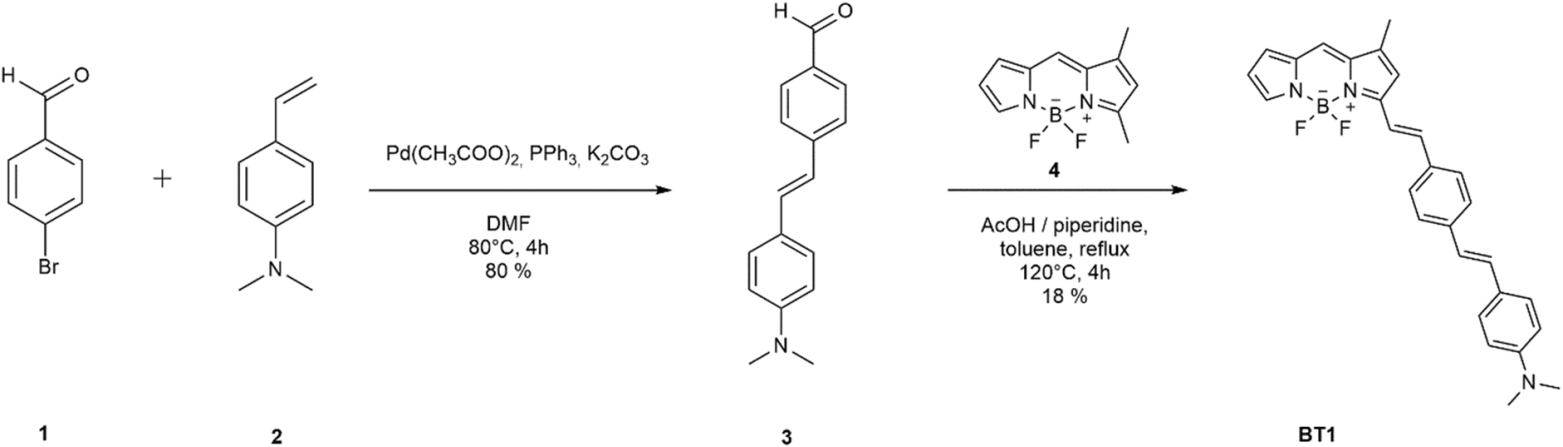
Two-step synthetic strategy for the preparation of BT1. (ChemDraw software version 20.1.0.112 (112); PerkinElmer Inc).

The BT1 compound was found to be the most promising compound as a selective marker of the P-tau protein NFTs in terms of *in silico* affinity, binding conformation and polarity. An efficient and versatile two-step synthetic strategy was developed (Fig. 2). BT1 was synthesized via a Knoevenagel condensation between the selected and commercially available BODIPY core (4) and the trans-4-[2-(4-dimethylaminophenyl) vinyl] benzaldehyde (3). The latter was synthesized by Heck reaction between 4-bromobenzaldehyde (1) and 4-dimethylaminostyrene (2), both commercially available, in the presence of a catalyst suitably chosen to promote the stereoselectivity of the reaction. The characterization of all new final products by ^1^H and ^13^C NMR, as well as ESI-MS, can be found in the Supplementary Information (Supplementary Figs. S11–S16).

**Figure 3:**
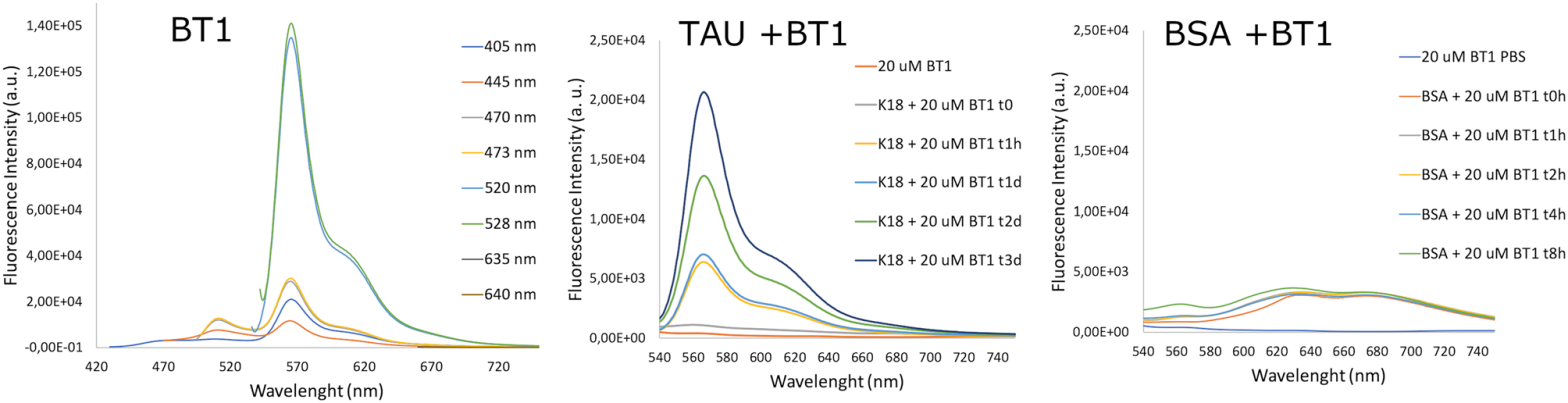
Fluorescence analysis of BT1. (**A**) Sample plot showing the emission spectra of synthesized BT1 (100 μM) in PBS buffer pH 7.4 and 1% DMSO according to the experimental conditions used for the immunostaining in human iPSC derived cortical neurons. The emission spectra were recorded at different λ_ex_ as indicated in the legend on the right. (**B**) Representative plot showing the time-course of BT1 (20 μM) (λ_ex_ = 520 nm) in the presence of unfibrillated K18 tau protein (70 μM) (t0) and fibrillated K18 upon induction with heparin (70 μM) after 1 h, 1 day, 2 days and 3 days at 37 °C. (**C**) representative graph reporting the time-course of fluorescence of BT1 (20 μM) (λ_ex_ = 520 nm) in the presence of unfibrillated BSA (20 μM) (t0) and fibrillated BSA upon heating at 62 °C after 1 h, 2 h, 4 h and 8 h. Note that only the presence of fibrillated TAU enhances BT1 fluorescence. (GIMP software "The GIMP Development Team, 2019. GIMP, URL: https://www.gimp.org).

BT1 has successfully dissolved up to 11 mM concentration in DMSO and accordingly diluted in the PBS buffer. Fluorescence emission bands of BT1 at a final concentration of 100 μM in PBS were analyzed and compared to the emission bands of the purchased fluorescent probe TAU1 (purity > 95%) in experimental conditions suitable for living cells experiments. In our hands, when dissolved in an aqueous solution, TAU1 showed an emission band at 510 nm when excited at a wavelength in the range 420–475 nm (Fig. S18). On the contrary, BT1, dissolved in the same buffer, showed a red-shifted intense band with a maximum at about 565 nm using an excitation wavelength at 520 nm (Fig. 3A). To confirm the specificity of BT1 for tau fibrils, fluorescence spectra of BT1 were analyzed in time course experiments in the presence of unfibrillated and fibrillated tau or Bovine Serum Albumin (BSA) proteins. Specifically, the incubation of K18 tau protein with heparin induced the formation of elongated tau fibrils, as confirmed by TEM images performed (see Supporting Information, Sect. 8.2 and Fig. S19). As shown in Fig. 3B, the fluorescence spectra of BT1 in the presence of preincubated tau fibrils displayed a strong enhancement at 565 nm indicating the BT1 binding to fibrillated tau. Parallel experiments were conducted monitoring BT1 fluorescence in the presence of BSA under experimental conditions known to induce the formation of BSA aggregates, characterized by the formation of β-sheet structures^45,46^. As reported in Fig. 3C BT1 fluorescence spectra did not significantly change over time in the presence of fibrillated BSA, thus indicating the selectivity of the BT1 probe for tau fibrils .

### Validation of human-induced pluripotent cell line WT#1 and neuronal differentiation

**Figure 4:**
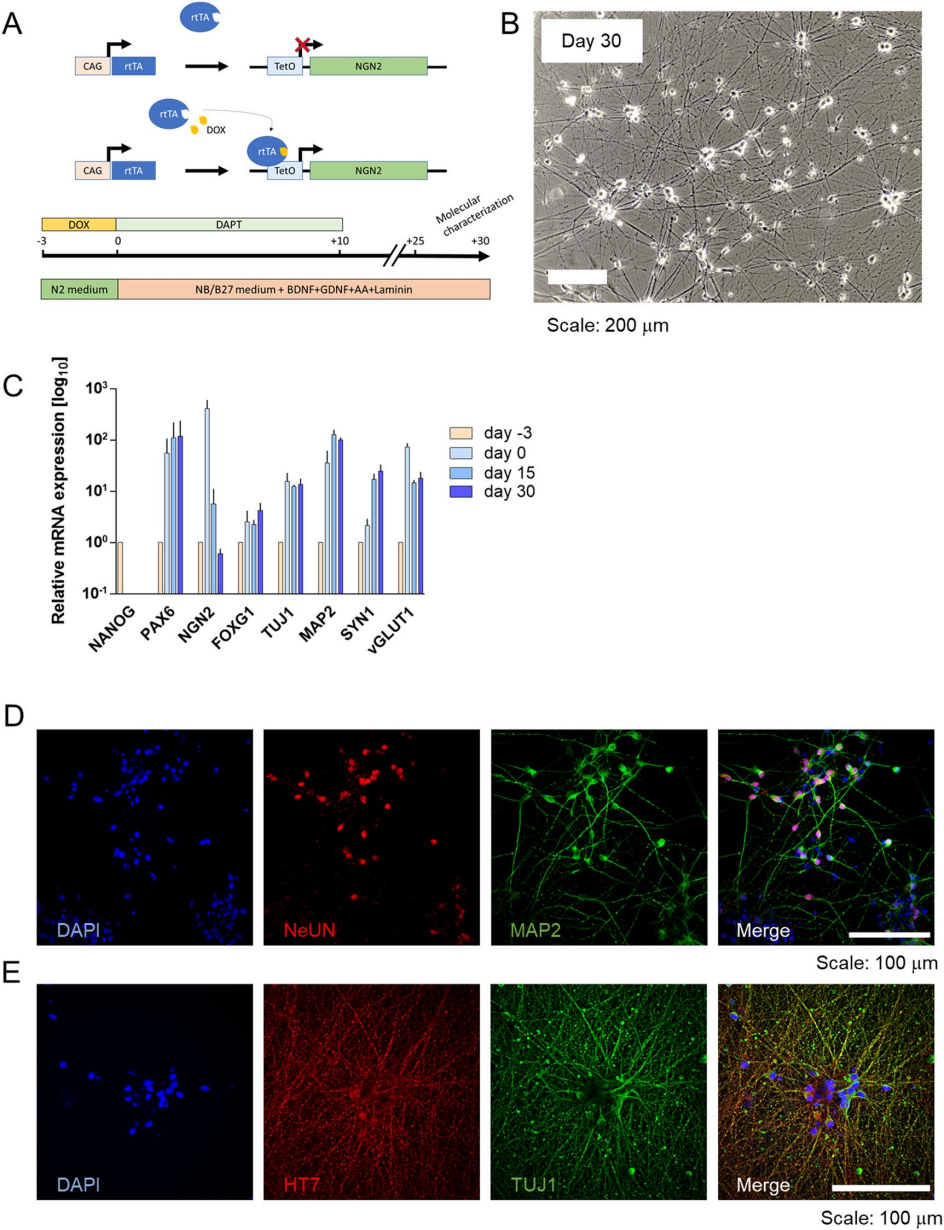
Molecular and cellular characterization of human iPSC derived NGN2 cortical neurons. (**A**) Schematic representation of the doxycycline-based strategy used for inducing the tuned expression of human NGN2 transcript. (**B**) Representative transmitted image of human iPSC-derived cortical neurons at day 30 in vitro. Scale bar 200 µm. (**C**) Real-time qRT-PCR analysis of specific molecular markers expression in human iPSC-derived cortical neurons at different time points of differentiation (n = 3 differentiation batches). (**D**) Representative images of immunostaining for NeUN (red), and MAP2 (green) expression in human iPSC-derived cortical neurons at day 30. Nuclei are stained in blue. Scale bar: 100 µm. (E) Representative images of immunostaining for total tau protein (HT7, red), and TUJ1 (green) expression in human iPSC-derived cortical neu-rons at day 30. Nuclei are stained in blue. Scale bar: 100 µm. (ImageJ software bundled with Java 1.8.0_172 software; URL: https://imagej.nih.gov/ij/).

An in-house iPSC line named WT#1 (Supplementary Fig. S17)^47,48^ was used for assessing the detection efficiency of tau aggregates by BT1 compound. The iPSC line WT#1 carries the doxycycline-inducible NGN2 cassette into the safe harbor AAVS1 locus^49^. NGN2-engineered iPSCs were treated for 4 days with 2 ug/mL doxycycline for inducing the rapid overexpression of the human NGN2 gene which is recognized as a potent molecular factor capable to drive the differentiation towards a neurocortical fate and specifically towards excitatory cortical neurons (Fig. 4A, B).

To confirm the proper differentiation of WT#1 line into neuronal cells after 30 days in culture (Fig. 4C), a time-course quantification by real-time PCR of the key neuronal molecular markers was performed. As expected, a steep increase of NGN2 transcript was found in the first week of culture as a consequence of the doxycycline treatment, together with the expression of PAX6 transcript, an early neural progenitor marker. Moreover, as maturation proceeded, NGN2 transcript level decreased in favor of dorsocortical and pan-neuronal markers FOXG1, TUJ1, MAP2. Excitatory synaptic transcripts (SYN and vGLUT1) arose in a time-dependent fashion between day 15 and day 30, suggesting the establishment of a mature neuronal network at later time points. At 30 days in culture, neuronal maturation was also evaluated at the protein level by confocal immunofluorescence analysis, confirming the expression of NeuN, TUJ1, and MAP2 neuronal markers (Fig. 4D) as well as T-tau expression (Fig. 4E), which showed the presence of a dense and ramified neuronal network.

### BT1 probe shows improved in vitro specificity for AT8-positive tau aggregates

With the aim to generate an *in vitro* model of tau hyperphosphorylation and neuronal degeneration, iPSC-derived NGN2 neuronal cultures were treated for 2 hours with 50 nM okadaic acid (OA), a potent inhibitor of many biological processes including the serine/threonine phosphatases, to induce protein hyperphosphorylation, and specifically to enhance the phosphorylation of endogenous neuronal tau protein. The effect of OA treatment was evaluated measuring the fluorescence signal intensity of both P-tau and oligomeric tau protein, two biological markers associated with AD related neuronal degeneration, using the specific AT8 and T22 antibodies respectively.

**Figure 5:**
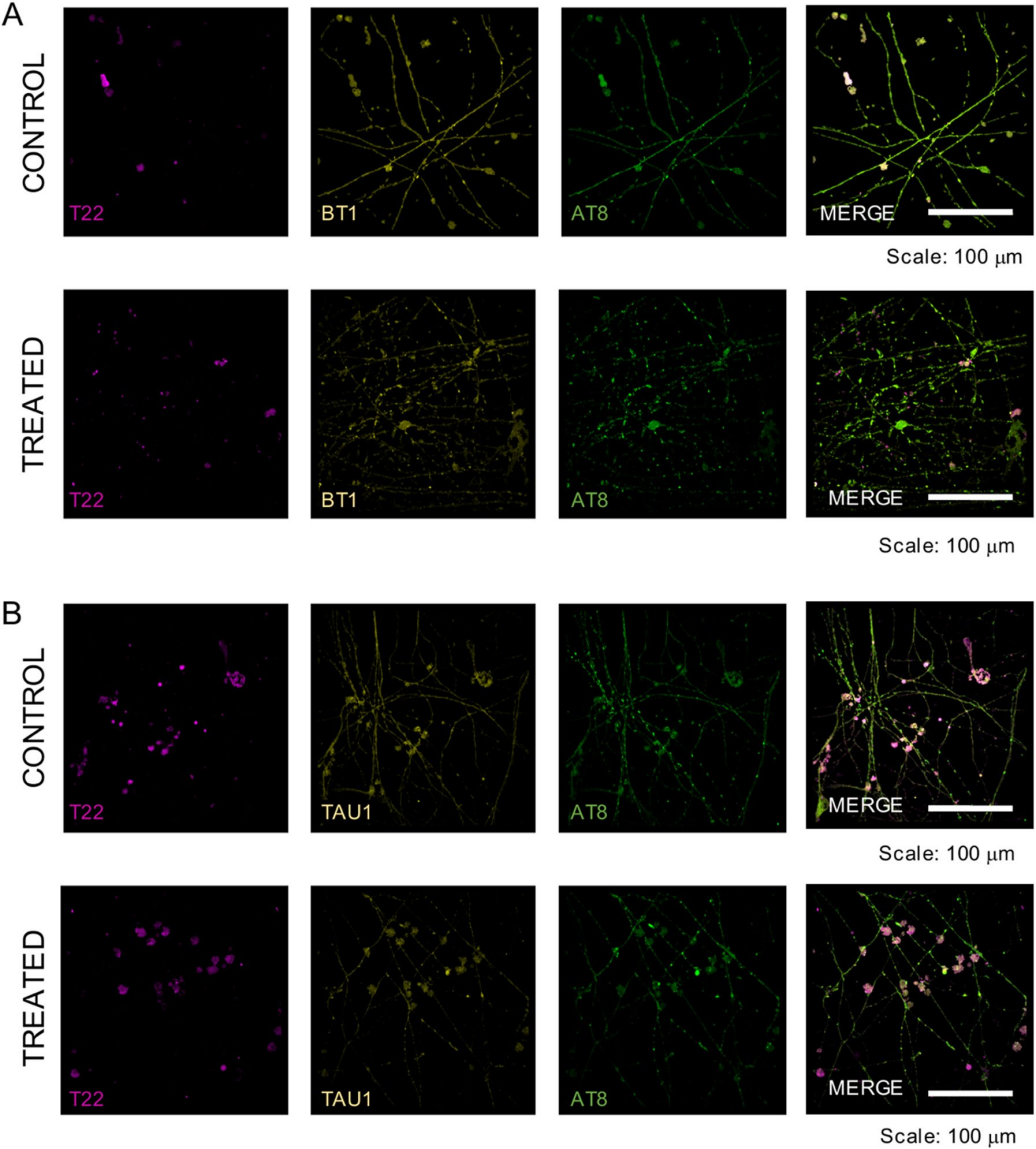
BT1 and TAU1 staining of hyperphosphorylated tau in iPSCs derived cortical neurons. (**A**) Top: Representative fluorescence images of control human iPSC-derived cortical neurons at 30 days in vitro incubated with BT1 (100 μM) for 30 min at 37 °C and then stained for AT8 (green) and T22 (magenta). Bottom: Representative fluorescence images of human iPSC-derived cortical neurons treated with okadaic acid (50 nM) for 2 h before incubation with BT1 (100 μM) for 30 min at 37 °C and relative staining for AT8 (green) and T22 (magenta). Images were acquired on an Olympus iX73 microscope equipped with an X-Light V3 spinning disc head using a 40 × magnification. Scale bar: 100 µm. (**B**) Top: Representative fluorescence images of control human iPSC-derived cortical neurons at 30 days in vitro incubated with TAU1 (100 μM) for 30 min at 37 °C and then stained for AT8 (green) and T22 (magenta). Bottom: Representative fluorescence images of human iPSC-derived cortical neurons treated with okadaic acid (50 nM) for 2 h before incubation with TAU1 (100 μM) for 30 min at 37 °C and relative staining for AT8 (green) and T22 (magenta). Images were acquired on an Olympus iX73 microscope equipped with an X-Light V3 spinning disc head using a 40 × magnification. Scale bar: 100 µm. (ImageJ software bundled with Java 1.8.0_172 software; URL: https://imagej.nih.gov/ij/).

To characterize the ability of BT1 to detect different forms of tau proteins, control and OA-treated neuronal cultures were incubated BT1 probe (100 μM; Fig. 5A) for 30 minutes at 37°C and then fixed and immunolabeled using AT8 and T22. Fluorescence signals were acquired through a spinning-disk confocal microscope equipped with conventional filters and a filter set specific for the spectra obtained for BT1 (excitation wavelength 520 nm, emission wavelength: 560/640 nm) at the concentration used on neuronal cultures (Fig. 3A; 4).

Parallel experiments were performed using the TAU1 probe (100 μM; Fig. 5B), acquired using the excitation and emission wavelengths reported in Supplementary Figure S18, in combination with AT8 and T22.

The acquired images were analyzed through a custom made MATLAB code capable of simultaneously performing image background correction, antibody dot-like signal detection and finally fluorescence intensity quantification and channel colocalization (see Methods section and Fig. 7).

**Figure 6:**
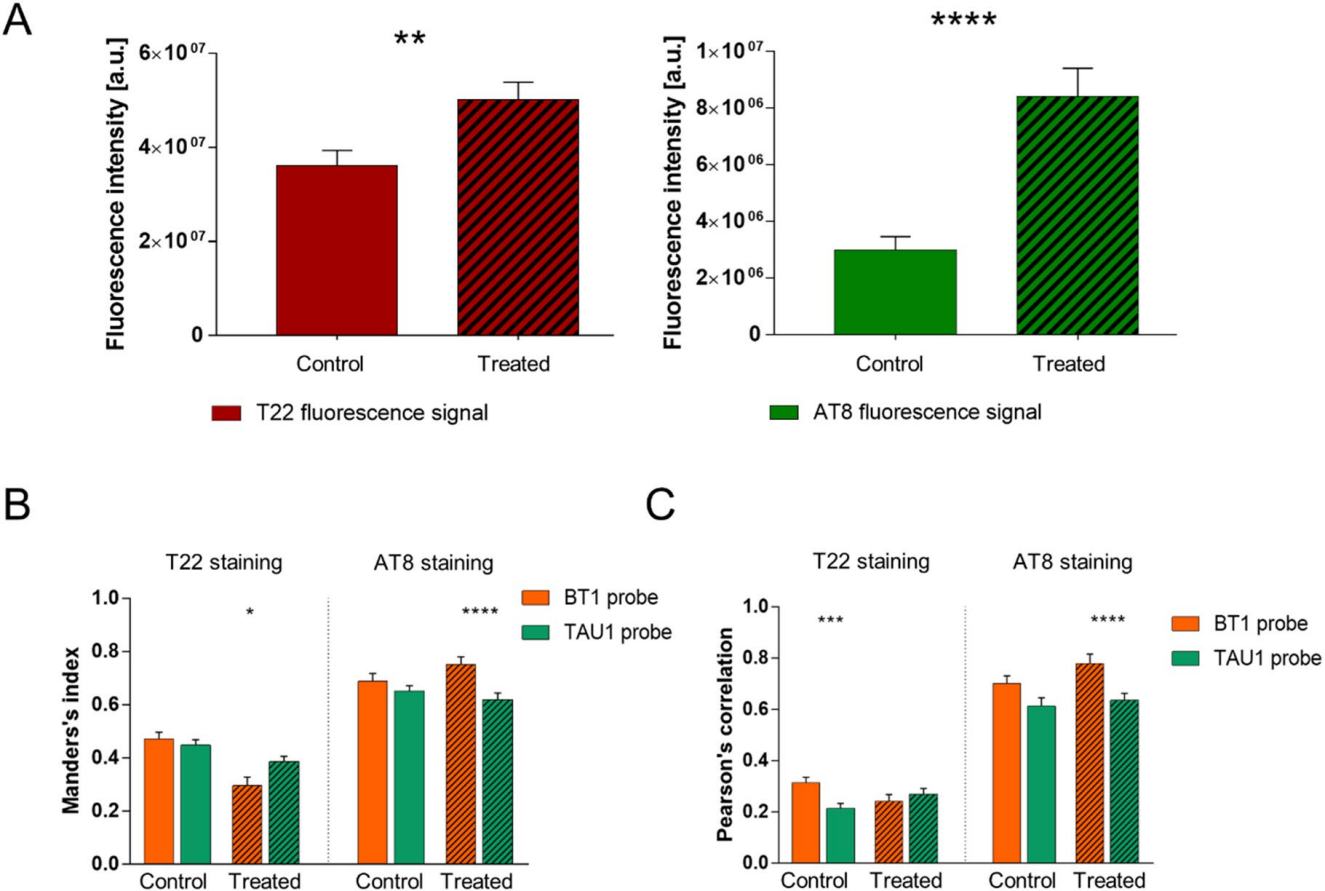
BT1 binds to hyperphosphorylated and oligomeric tau in OA treated neurons. (**A**) Bar charts showing the fluorescence intensity quantification of (left) T22 signal (**p = 0.002, MW test; n = 53/3, fields of view/batches) and (right) AT8 signals (****p < 0.0001, MW test; n = 53/6/3, fields of view/batches) in control condition and after the treatment with okadaic acid (50 nM) for 2 h. (**B**) *Left*, Manders’s colocalization in-dex of T22 staining with TAU1 (green) and BT1 (orange) fluo-rescence signal in control condition (p = 0.48, t-test; n = 25/3, fields of view/batches) and after the treatment with okadaic acid (50 nM) for 2 h (*p < 0.017, MW test; n = 25/3, fileds of view/batches). *Right*, Manders’s colocalization index of AT8 staining with TAU1 (green) and BT1 (orange) fluorescence signal in control condition (*p* = 0.408, MW test; n = 25/3, fields of view/batches) and after the treatment with okadaic acid (50 nM) for 2 h (****p < 0.0001, MW test; n = 25/3, fileds of view/batches), as determined using the custom-made MATLAB code. (**C**) On the left, Pearson’s correlation index of T22 staining with TAU1 (green) and BT1 (orange) fluorescence signal in control condition (***p = 0.0008, t-test; n = 25/3, fields of view/batches) and after the treatment with okadaic acid (50 nM) for 2 h (*p* = 0.423, t-test; n = 25/3, fileds of view/batches). On the right, Pearson’s correlation index of AT8 staining with TAU1 (green) and BT1 (orange) fluorescence signal in control condition (*p* = 0.056, MW test; n = 25/3, fields of view/batches) and after the treatment with okadaic acid (50 nM) for 2 h (****p < 0.0001, MW test; n = 25/3, fileds of view/batches), as determined using the custom-made MATLAB code. (Matlab software, version 2021a; URL: https://it.mathworks.com/products/matlab.html?s_tid=hp_products_matlab).

As shown in Fig. 6A, OA treatment increased the expression of both P-tau and oligomeric tau in human iPSC-derived neurons, confirming the establishment of a neurotoxic condition within the neuronal networks. The quantified data, reported in Fig. 6, showed that both BODIPY based probes bind to P-tau and oligomeric tau, as shown by colocalization with AT8 and T22 respectively. Strikingly, BT1 displayed improved detection of AT8-positive P-tau tangles when compared to TAU1, as demonstrated by the higher Mander’s (Fig. 6B) and Pearson’s (Fig. 6C) correlation indexes in OA treated human neurons.

## Discussion

We here report the design and synthesis of a novel BODIPY based fluorophore developed to bind hyperphosphorylated and oligomeric tau, and its characterization *in vitro* and in a humanized model of cortical neurons.

We combined synthetic organic chemistry and computational modelling, biochemistry and molecular and cellular biology in a concerted multidisciplinary strategy to developed fluorescent sensors for selectively targeting neurofibrillary tangles (NFTs) of the tau protein which could represent a valuable tool in Alzheimer’s disease (AD) diagnosis. The BODIPY core emerged as privileged scaffold in the design of cross β-sheet fluorescent binders, based on molecular modeling predictions and prior studies. Here, we designed a small-size focused library of fluorescent probes consisting of a BODIPY core (electron acceptor) featuring a highly conjugated system ending with an aliphatic amine (electron donor) with a length in the range 13-19 Å, and characterized by a different polarity. This structural modification was rationally designed to improve the P-tau vs Aβ selectivity respect to the previously reported TAU1, that has been shown to bind also Aβ^50^, and the small-size focused library was docked toward the 6-mer model of the PHF6 fragment, present in the R3 region of the P-tau protein and responsible for the propensity of the protein itself to assemble into fibrils. Preliminary docking simulations were carried out to monitor the ability of designed compounds to fit the target binding site. Even though the BT2 compound provided the better theoretical binding affinity among the eight new BODIPY probes here designed (BT1-8), it presented two statistically most relevant docking poses in which the BODIPY core binds with opposite orientation near the entrance of the lipophilic tunnel. Moreover, BT2 displayed significantly lower solubility in buffers suitable for the use of a tau probe in living cells, tissues or organisms compared to BT1. Thus, combining the predicted affinity and binding mode with the polarity, we decided to synthesize BT1 as a model compound of the series for further investigations.

The probe BT1 was prepared via an efficient and cost-effective two-step synthetic strategy and exhibited excellent photophysical properties. The probe was clearly able to efficiently provide *in vitro* binding of fibrillated tau protein filaments with prominent change in fluorescence intensity and good selectivity, as demonstrated by the lack of fluorescence increase observed in parallel experiment performed with fibrillated BSA.

We also here report that the designed BODIPY based BT1 compound improved the detection of P-tau, oligomeric tau and thus potentially tau tangle formation in living human cortical neurons, when compared to the reference probe TAU1^37^.

Specifically, we demonstrated the ability of BT1 to bind P-tau and oligomeric tau in a model system based on human iPSCs derived NGN2 cortical neurons. Indeed, BT1 staining displayed a good colocalization with conventional antibodies against the Ser202 and Thr205 phosphorylated tau (AT8)^51^ and tau oligomers (T22)^52^ on iPSC derived human cortical neurons treated with okadaic acid, to promote endogenous tau hyperphosphorylation.

We characterized the ability of BT1 to bind phosphorylated and oligomeric tau using an *in vitro* humanized cellular model that represents a physiologically relevant cellular platform to study neuronal function in homeostatic and pathological condition. Indeed, as here reported, using molecular and confocal innunofluorescence characterization, this cellular system mimic the development of dorsocortical neurons expressing, in a time dependent fashion, neuronal markers (NeuN, FOXG1, TUJ1 and MAP2), excitatory synaptic transcripts (SYN and vGLUT1) as well as T-tau, with the formation of dense and ramified neuronal networks.

Accordingly with numerous studies, *in vitro* models obtained from human iPSCs represent an important tool to dissect molecular pathological mechanisms, to identify novel therapeutic targets, and to test drug candidates. Indeed, the improvement in human somatic cells reprogramming into iPSCs, and in protocols for iPSCs differentiation into brain cells is paving the road for a deeper analysis of development and functions of the human brain in physiological and pathological conditions. Moreover, for further investigations, the availability of patient-derived and gene edited iPSCs, displaying the genome and the molecular phenotype of the affected individuals, allows to go beyond the limitation of transgenic mouse models, and to develop in 2D and 3D *in vitro* cultures more faithful disease models to investigate the pathology at a cellular and molecular level^53,54^.

Notably, in our experimental conditions we did not observe any toxicity or change in cellular viability using BT1 on living hiPSCs derived cortical neurons. It has to be considered, however, that our data rely on a single BT1 concentration, that provided a convenient balance between a BT1 spectrum suitable for co-immunostaining with different tau antibodies and a DMSO concentration fitting with viability of neurons and iPSCs viability^55^.

It has to be noticed that while previous reports^37,50^, indicated that the emission wavelength of TAU1 is >650 nm, prior to perform measurements on iPSC derived cortical neurons we measured excitation/emission spectra for both BT1 and TAU1 using the experimental conditions suitable for neuronal tau imaging and we found different results. This discrepancy may arise from different solution buffer conditions. Indeed, we measured excitation/emission spectra of TAU1, first dissolved in 100% DMSO and then diluted in PBS to reach a final concentration of 100 μM (DMSO 1%), suitable to be used in living cells. In these condition no peaks were detected at 660 nm (see Supplementary Fig. 18), a wavelength reported for TAU1 dissolved in chloroform and in the presence of tau protein^37,50^. Indeed, the observed change in the TAU1 spectrum may arise from the choice of solution characterized by different polarity (from apolar solvent such as chloroform to polar solution as DMSO + H_2_O).

In conclusion, the optimized BT1 compound can be used to detect phosphorylated and oligomeric tau on human living tissue. Accordingly, BT1 will be employed as a model compound to design a novel congeneric series of promising tau probes following deeper simulations to compute energy contributions.

Moreover, given the chemical versatility of the BODIPY scaffold, owning to their relative ease of substitution and generally highly fluorescent nature, our findings offer new directions into the structural optimization of specific compounds for different target proteins, helping in the differential diagnosis of neurodegenerative diseases associated with the deposition of protein aggregates.

## Methods

### Materials

Compound TAU1 was purchased from SYNCOM (custom synthesis) and used without further purification. The chemical identity of compounds was assessed by re-running Nuclear Magnetic Resonance spectroscopy (NMR) experiments and proved to be in agreement with the literature data reported for this compound. The purity, checked by reversed-phase High Performance Liquid Chromatography (HPLC), was approximately 95%. When not specified reagents and solvents were purchased from commercial suppliers (MERCK Life Science, TCI Chemicals, Eurisotop) and were used without further purification. UV/vis spectra were recorded on a Jasco V-750 spectrophotometer and fluorescence spectra were obtained using a Shimadzu RF-6000 spectrofluorophotometer. Melting points were recorded with Büchi melting point B-545 apparatus in open capillaries and are not corrected. NMR spectra have been acquired with a Bruker Avance/Ultra ShieldTM 400 spectrometer operating at 400.13 MHz for ^1^H and 100.62 MHz for ^13^C at room temperature, using tetramethylsilane (TMS) as internal standard and 5 mm diameter glass tubes. Chemical shifts (δ) are reported in parts per million (ppm) and coupling constants (J) in hertz (Hz), approximated to 0.1 Hz. The residual solvent peak was used as an internal reference for ^1^H and ^13^C NMR spectra and is referenced to CD_2_Cl_2_ (δ= 5.26 ppm for ^1^H, δ=53.84 ppm for ^13^C). Data for ^1^H NMR are reported as follows: chemical shift, multiplicity (br = broad, ovrlp = over-lapped, s = singlet, d = doublet, t = triplet, q =quartet, m = multiple, dd = doublet of doublets), coupling constant, integral. All ^13^C NMR spectra were obtained with complete proton decoupling. Spectra were processed with the program MestReNova version 12.0.0-20080, FT and zero filling at 64K. Chromatography was carried out on 60 Å silica gel (40–63 µm, 230–400 mesh). All reactions were monitored by thin-layer chromatography (TLC), and 60 Å silica gel on TLC plates were used. The compounds on TLC were revealed by quenching fluorescence at 365 nm using a 4W UV lamp because the fluorescent markers of the invention are characterized by an excitation wavelength of 350–650 nm and an emission wavelength of 390–800 nm. Electron spray ionization mass spectra (ESI-MS) were per-formed on Bruker BioApex Fourier transform ioncyclotron resonance (FT-ICR) mass spectrometer.

### Molecular modelling

Ligands were designed in 2D with PICTO version 4.4.0.4 (OpenEye Scientific Software, Santa Fe, NM)^56^ and converted into 3D format by OMEGA version 3.1.0.3 (OpenEye Scientific Software, Santa Fe, NM)^57,58^. The most prevalent ligand protonation form at pH 7.4 was assigned by QUACPAC version 2.0.0.3 (OpenEye Scientific Software, Santa Fe, NM)^59^, while energy minimization was carried out by SZYBKI version 1.10.0.3 (OpenEye Scientific Software, Santa Fe, NM) 44 using the MMFF94S force field^60^. CLogP was calculated with ChemDraw (PerkinElmer Informatics, Inc). The 6-mer model of the most conserved channel formed by four adjacent β-sheets was prepared as described in Verwilst et al. ^37^ starting from the PDB-ID 5K7N^61^. Molecular docking was carried out with AutoDock4.2 using default settings^62^. Since common molecular modeling software such as OpeEye and AutoDock do not provide force field parameters for boron atoms, in our study it was replaced by a carbon atom having sp^3^ hybridization.

### Synthesis of 3

Trans-4-[2-(4-dimethylaminophenyl)vinyl]benzaldehyde was synthesized via Heck reaction starting from 4-bromobenzaldehyde (1) and 4-dimethylaminostyrene (2) according to a modified literature procedure^63^. The suitable catalyst, chosen to promote the stereoselectivity of the reaction, was prepared in situ: 16.8 mg of palladium acetate (II) (0.075 mmol) and 19.7 mg of triphenylphosphine (0.075 mmol) have been soluble in DMF. After 10 minutes, a solution of 202 mg of 4-bromobenzaldehyde 1 (1.5 mmol), 264.6 mg of 4-dimethylminostyrene 2 (1.8 mmol) and 414 mg of potassium carbonate (3.00 mmol) in 3 mL of DMF has been added to the catalyst solution. The reaction was left in agitation at 80 °C for 4 h, after which 50 mL of a saturated NH_4_Cl solution in water was added. Later, the mixture was extracted with CH_2_Cl_2_ (3 × 100 mL) and the organic layers were combined, dried with Na_2_SO_4_ anhydrous and concentrated under vacuum. Compound 3 (1,074 mmol) was obtained by cold hexane crystallization as a yellow solid (270 mg, 72%). Mp: 218.0–220.0 °C. ^1^H NMR (400 MHz, CH_2_Cl_2_) δ 9.94 (s, 1H), 7.82 (d, J = 8.3 Hz, 2H), 7.62 (d, J = 8.3 Hz, 2H), 7.45 (d, J = 8.8 Hz, 2H), 7.23 (d, J = 16.3 Hz, 1H), 6.96 (d, J = 16.3 Hz, 1H), 6.72 (d, J = 8.8 Hz, 2H), 3.00 (s, 6H). ^13^C NMR (101 MHz, CH_2_Cl_2_) δ 191.3, 150.8, 144.5, 134.6, 132.4, 130.1, 128.1, 126.1, 124.5, 122.4, 112.3, 79.5, 40.1. ESI-MS(m/z): [M+H]+ calcd. For C_17_H_18_NO, 252.13; found 252.17.

### Synthesis of BT1

A solution of 100 mg (0.45 mmol) of commercially available 1, 112.95 mg (0.45 mmol) of compound 3, in the presence of 0.35 mL of acetic acid (3.5 mmol) and 0.35 mL of piperidine (6.12 mmol) in 10 mL of toluene was heated under Dean−Stark conditions for 4 h. The reaction was allowed to cool to room temperature, and 50 mL of a saturated aqueous NH_4_Cl solution was added. The mixture was extracted with CH_2_Cl_2_ (3 × 100 mL), and the organic layers were combined, dried over Na_2_SO_4_ anhydrous and concentrated at reduced pressure. Column chromatography (silica, EtOAc/hexane, 1:9 → CH_2_Cl_2_) resulted in 18 mg (0.04 mmol,18%) of BT1 as black solid. Mp: 257-262 °C. ^1^H NMR (400 MHz, CH_2_Cl_2_) δ 7.63-7.57 (m, 4H), 7.53 (d, J = 8.4 Hz, 2H), 7.46–7.42 (m, 3H), 7.23 (s, 1H), 7.16 (d, J = 16.4 Hz, 1H), 6.97–6.91(m, 2H), 6.83 (bs, 1H), 6.72 (d, J = 8.9 Hz, 2H), 6.48-6.47 (m, 1H), 2.99 (s, 6H), 2.34 (s, 3H). ^13^C NMR (101 MHz, CH_2_Cl_2_) δ 151.4, 149.8, 141.2, 141.0, 139.0, 134.9, 131.3, 129.3, 128.9, 128.7, 127.2, 126.4, 125.9, 123.9, 123.6, 121.3, 118.2, 118.1, 117.0, 113.1, 41.0, 12.1. ESI-MS(m/z): [M+H]+ calcd. For C_28_H_27_BF_2_N_3_, 453.22; found 454.33.

### Tau and BSA protein fibrillation

K18 domain of tau protein was purified as described in the supplementary 8.1 section. Fibrils of purified recombinant tau protein were prepared by incubating different concentrations of tau (typically in the range of 20–100 μM) at 37 °C in PBS, pH 7.4, containing the anionic cofactor heparin (Bio-Rad MW 6000) in the ratio of 1:1 heparin to protein. Incubation time varied between 1 hour up to 76 hours. The optimal experimental conditions for the formation of stable fibrils were ascertained by thioflavine S fluorescence assay and transmission electron microscopy (see Section 8.2 and Fig. S19).

The formation of ordered β-sheet-based aggregates of BSA was induced by heat treatment at 62 °C for 1h, 2h, 4h and 8 h of unfibrillated BSA as previously reported^45,46^.

### Fluorescence analysis of BT1

BT1 probe was dissolved in 11 mM in DMSO and then diluted in PBS buffer pH 7.4 at a final concentration of 100 μM and 1% DMSO in order to mimic the *in vitro* experimental conditions. Different excitation wavelengths, in the range 405–640 nm, were selected and the corresponding emission spectra were recorded as shown in Fig. 3A with a RF-6000 Shimadzu (Shimadzu Corporation).

Time-dependent fluorescence of BT1 in the presence of protein tau fibrils, was performed using the aggregation-promoting conditions assessed. Fluorescence was measured in a range from 540 to 750 nm with an excitation wavelength of 520 nm in a 1 cm path cuvette. Measurements were performed at room temperature by adding 20 μM BT1 to unfibrillated and fibrillated tau or BSA proteins. Fluorescence was measured in a range from 540 to 750 nm with an excitation wavelength of 520 nm in a 1 cm path cuvette.

### Human iPSCs Maintenance

Induced pluripotent stem cell line (WT#1)^47,48^ was maintained in mTeSR Plus medium (STEMCELL Technologies) on growth factor‐reduced Matrigel-coated (Corning; dilution 1:100) plates at 37 °C in 5% CO_2_ and iPSC colonies were passaged with PluriS-TEM Dispase-II (Merck Life Science) when 80% confluent.

### iPSCs differentiation to cortical neurons

iPSC WT#1 was differentiated with a two-step protocol based on doxycycline-induced expression of human NGN2 transcript as previously described. Briefly, human iPS cells were treated with 1X Accutase (Thermo Fisher Scientific) and plated onto growth factor-reduced Matrigel-coated plates at a density of 1000 cells/mm^2^ in mTeSR Plus containing 10 μM Rock-inhibitor Y-27632 (STEMCELL Technologies) and 2 μg/mL doxycycline (Merck Life Science). The day of seeding is set as day minus 3 (D-3). One day after seeding (D-2), the medium is switched to N2 medium consisting of DMEM/F12 [1:1], 1% N2 supplement, 1% NEAA, 1% GlutaMAX (Thermo Fisher Scientific) supplemented with 2 μg/mL doxycycline (Merck Life Science) to sustain hu-man NGN2 expression. N2 medium was refreshed every day. Three days after (D0), the early born neurons were dis-sociated with Accutase and plated onto PDL/laminin-coated (Merck Life Science) dishes at a density of 500 cells/mm2 in maturation medium consisting of Neurobasal, 2% B27 with vitamin A, 1% GlutaMAX (Thermo Fisher Scientific), 0,5 μg/mL laminin (Merck Life Science), 20 ng/mL BDNF (Peprotech), 20 ng/mL ascorbic acid (Peprotech), 10 ng/mL GDNF (Peprotech) supplemented with 2 μg/mL doxycycline, 10 μM Rock-inhibitor Y-27632 and 10 μM DAPT (STEMCELL Technologies). After 24 hours, doxycycline and Y-27632 were removed and the medium was refreshed every three days until day 10. Optionally, 2 μM Ara-C (Merck Life Science) was added to the medium on day 4 and day 7 to remove proliferative cells. Thereafter, the maturation medium was half changed weekly until the experimental window was reached around D30.

### RT-PCR and RT-qPCR

Total RNA was extracted with the EZNA Total RNA Kit I (Omega Bio-Tek) and retrotranscribed using the iScript Reverse Transcription Supermix for RT-qPCR (Bio-Rad). Real-time RT-PCR was performed with iTaq Universal SYBR Green Supermix (Bio-Rad) on a ViiA 7 Real-Time PCR System (Applied Biosystems) and housekeeping gene ATP5O (ATP synthase, H+ transporting, mitochondrial F1 complex, O sub-unit) was used as an internal control. A complete list of primers is provided in the supplementary material (Supplementary Table 3).

### Induction of a tau hyperphosphorylated state and staining with BODIPY-base probes

iPSC-derived cortical networks were treated with 50 nM Okadaic acid (OA; Merck Life Science) for 2 hours at 37 °C in 5% CO_2_. Afterward, untreated and treated neuronal cultures were incubated with either 100 μM TAU1 probe or 100 μM BT1 probe for 30 minutes at 37 °C and thus fixed for 15 minutes at room temperature with cool and fresh-made 4% PFA. Note that we did not observed neurotoxicity when using 100 µM BT1 and TAU1.

### Immunostaining

Fixed iPSC-derived cortical neurons were permeabilized with 0.2% Triton X-100 (Merck Life Science) in 1X TBS and incubated for 1 hour in blocking solution containing 1X TBS, 0.2% Triton X-100, and 5% goat serum (Merck Life Science). Afterwards, the cells were incubated in blocking solution containing primary antibodies overnight at 4°C. The primary antibodies employed in this study were anti PHF-tau Ser202/Thr205 (AT8; dilu-tion 1:200; Thermo Fisher Scientific) and anti-oligomeric tau (T22; dilution 1:200; Merck Life Science) followed by incubation with secondary antibody (dilution 1:1000) for 1 hour at room temperature. A complete list of antibodies is provided in the supplementary material (Supplementary Table 2).

Images were acquired with an inverted microscope equipped with the X-Light V2 Spinning Disk Confocal module (Crest Optics) with a 40×/NA 0.75 objective lens in stack with z-step of 0.4 μm. Image processing was per-formed through custom numeric codes implemented in MATLAB environment.

### Image analysis

Image processing was performed through custom numeric codes implemented in MATLAB environment. The algorithm consists of three main steps: image correction, image binarization and colocalization.

**Figure 7:**
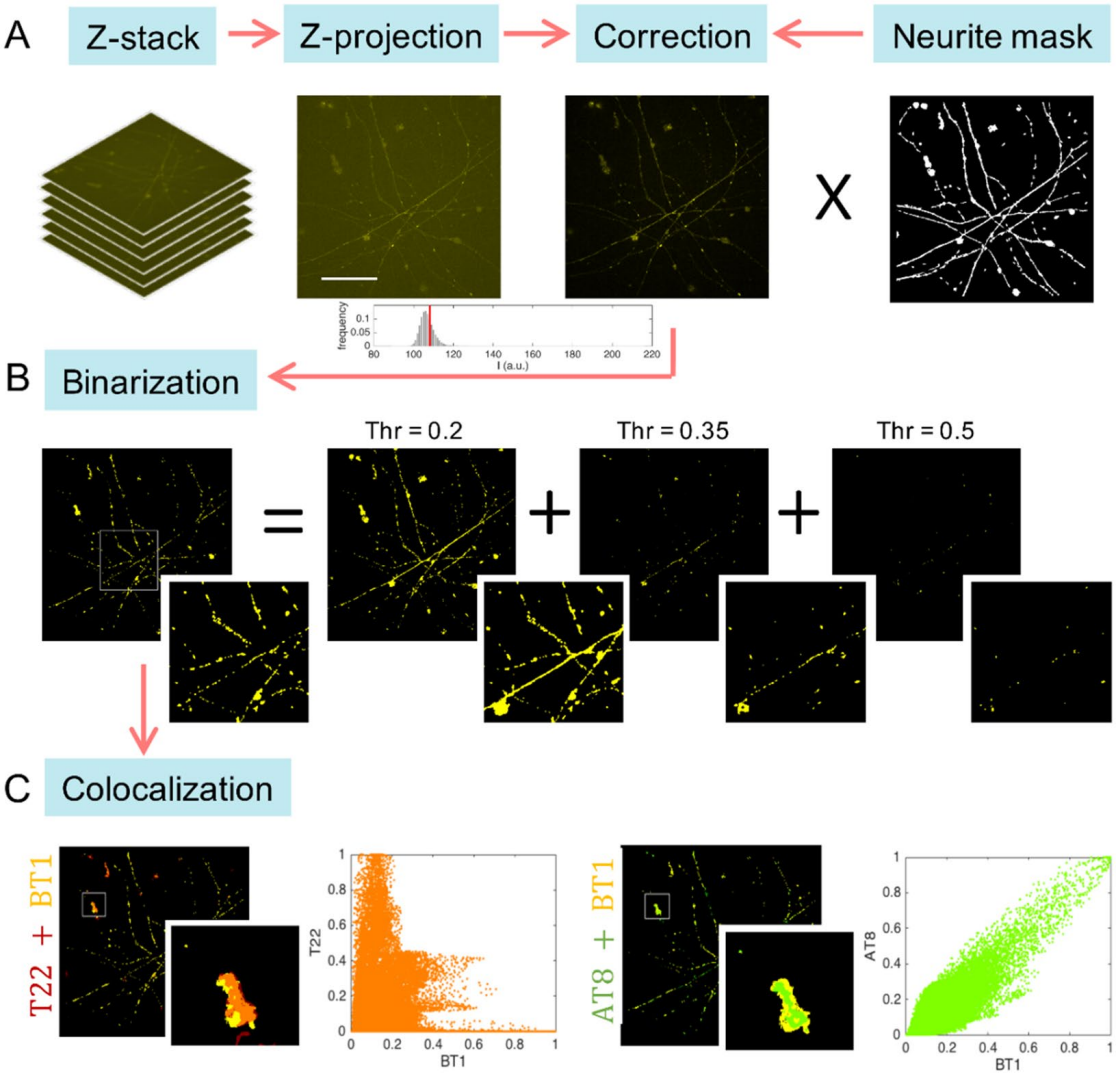
Schematic representation of MATLAB based algorithm for image analysis. (**A**) The maximum z-projection is calculated for each z-stack, then the background level is automatically retrieved and subtracted. The neurite structure mask is de-fined and applied as a filter to exclude unwanted signals located not in neurite structures. (**B**) An iterative thresholding procedure is used to binarized the image. Starting from the threshold value Thr = 0.2 (20% of the maximum signal) at each iteration, the threshold limit is increased by 0.15 units. The entire threshold image collection is combined to get a well-resolved binarized image. (**C**) The colocalization of the probe (BT1) with the antibodies (T22 and AT8) is calculated. Two well-known colocalization methods are exploited: the scatter plots (orange and green), which allow visualizing the correlation measured by PC coefficient, and the merged binary images (red/yellow and green/yellow), which allow to visualize the co-occurrence measured by M1 coefficient. (Matlab software, version 2021a; URL: https://it.mathworks.com/products/matlab.html?s_tid=hp_products_matlab).

*Correction* (Fig. 7A) For each channel, the maximum z-projection obtained from the z-stack was examined. Noise and background affecting each channel were removed before carrying out the quantitative analysis. A moving average filter (5x5 pixels window) was used to reduce the noise, the background affecting each channel was automatically retrieved and subtracted through the statistical analysis of global pixel intensity distribution (negative values have been set to zero). More precisely, a histogram shape-based method was used, which identifies background levels beyond the peaks of the smoothed histograms. Furthermore, to exclude unwanted signals, a neurite structure mask, obtained by thresholding pixel values on the AT8 channel, was applied to each channel.

*Binarization* (Fig. 7B) Meaningful pixels were selected for each corrected image. For this purpose, removing background and thresholding, unfortunately, was not sufficient: low threshold values lead to an overestimation of the signal, and higher values could cause information loss. To overcome this problem, the following iterative procedure was designed. Starting from a low threshold value, 20% of the maximum signal, at each step the threshold was increased by 15%, until enough pixels were no longer selected. A different binary image was obtained for each threshold: the final binary image was obtained by combining them and deleting the largest of the overlapping areas (more than 70% overlapping). Finally, the area covered by the fluorescence signal and the corresponding integrated density were calculated for each channel.

*Colocalization* (Fig. 7C) To quantify the colocalization of the probe with the anti-bodies T22 and AT8 the following procedure was followed. Two of the most used colocalization coefficients were exploited: Pearson’s correlation coefficient (PC) and Manders’ overlap coefficient (M1), which measure correlation and co-occurrence respectively. An easy way to visualize the dependence of pixels in dual-channel images is to consider a pixel distribution diagram called scatter plot or fluorogram (orange and green plots in Fig. 7C), where x-coordinates are given by the pixel intensities of the probe image and y-coordinates are given by the pixel intensities of the antibody image. This scatter plot provides the first intuitive evidence of colocalization: in a complete colocalization the points on the diagram are distributed around a line (e.g. green plot in Fig. 7C) and the spread with respect to the line is measured by the correlation coefficient PC. More precisely, PC quantifies pixel intensity spatial correlation and, being calculated on corrected not binary images, it is independent of the binarization step. However, in a more general scenario (e.g. orange plot in Fig. 7C), in addition to scatter plots and PC it is necessary to evaluate colocalization by using the corrected binary images and calculating the coefficient M1. M1 is defined as the ratio of the ‘summed intensities of pixels from the antibody image for which the intensity in the probe channel is above zero’ to the ‘total intensity in the antibody channel’. Hence M1 measures the fraction of antibody signal coinciding with probe signal, an ideal tool to quantify probes efficiency. PC and M1 can be considered reliable colocalization coefficients since images were properly corrected, similar acquisition and thresholding conditions were applied, and a large set of images was compared.

### Statistical data analysis

Statistical analysis, graphs and plots were generated using GraphPad Prism 6 (GraphPad Software) and MATLAB 2016b (MathWorks). To verify whether our data sets were reflecting normal distribution, the Shapiro-Wilk normality test was performed. Where the normality distribution was not fulfilled, statistical significance analysis was performed using the non-parametric two-sided Mann–Whitney test (MW test, *P*=0.05). In all other cases, whether not stated otherwise, t-Student test (*P*=0.05) was performed, and data set are given as mean ± standard error of the mean (s.e.m.).

## Supplementary Information

Supplementary Information.

## Data Availability

The data that support the findings of this study are available from the corresponding author upon reasonable request.
