# Supplementary material for: Rational design and synthesis of a novel BODIPY-based probe for selective imaging of tau tangles in human iPSC-derived cortical neurons
Source: Sci Rep. 2022 Mar 28;12:5257. doi: 10.1038/s41598-022-09016-z (PMC8960764; doi:10.1038/s41598-022-09016-z)
Supplement: Supplementary file 1 — Supplementary Information. [file 41598_2022_9016_MOESM1_ESM.docx]

**Supplementary Material**

**Rational design and synthesis of a Novel BODIPY-based probe for selective imaging of Tau Tangles in human iPSC-derived cortical neurons**

Alessandro Soloperto, Deborah Quaglio, Paola Baiocco, Isabella Romeo, Mattia Mori, Matteo Ardini, Caterina Presutti, Ida Sannino, Silvia Ghirga, Antonia Iazzetti, Rodolfo Ippoliti, Giancarlo Ruocco, Bruno Botta, Francesca Ghirga, Silvia Di Angelantonio, Alberto Boffi

1. **Additional computational data**

**Figure S1**

**Figure S1.** Chemical structures of compounds BT2-BT8. (ChemDraw version 20.1.0.112 (112); PerkinElmer Inc)

**Table S1**

**Table S1.** Predicted Binding affinity of compounds BT1-BT8.

- 1. Docking complexes

**Figure S2**


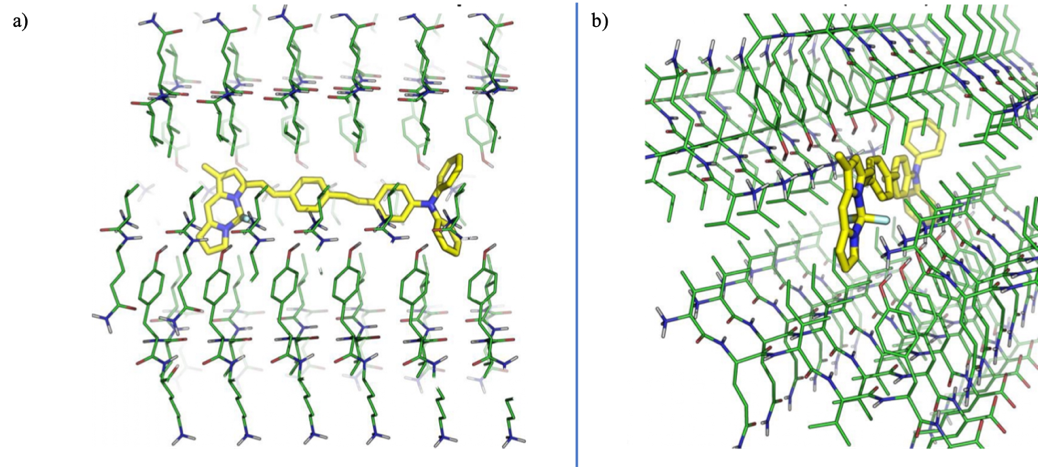


**Figure S2.** Molecular docking of BT2 pose 1 against the crystallographic structure of the PHF6 fragment. (a) Side view of the molecule in the protein tunnel. (b) Diagonal view of the molecule in the protein tunnel. (PyMOL Software. The PyMOL Molecular Graphics System, Version 2.2.0 Schrödinger, LLC. URL:https://pymol.org/2/)

**Figure S3**


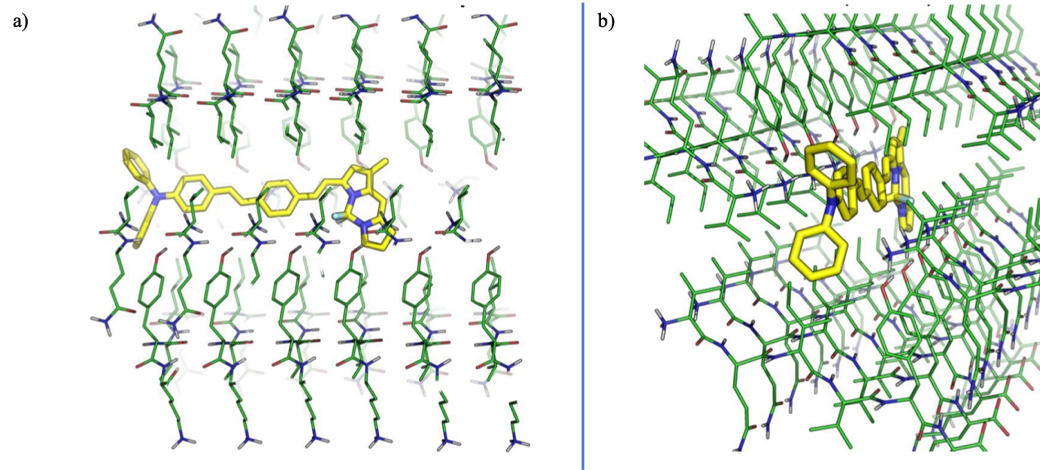


**Figure S3.** Molecular docking of BT2 pose 2 against the crystallographic structure of the PHF6 fragment. (a) Side view of the molecule in the protein tunnel. (b) Diagonal view of the molecule in the protein tunnel. (PyMOL Software. The PyMOL Molecular Graphics System, Version 2.2.0 Schrödinger, LLC. URL: https://pymol.org/2/)

**Figure S4**


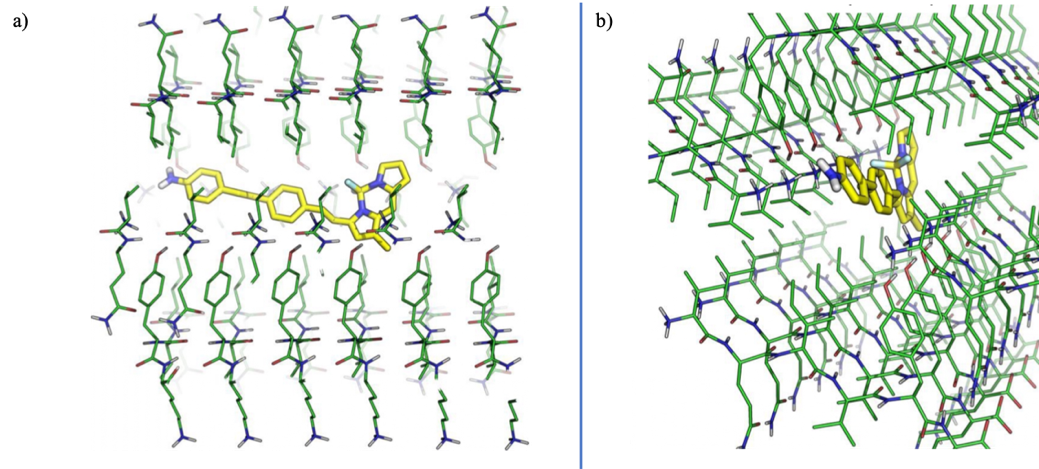


**Figure S4.** Molecular docking of BT3 against the crystallographic structure of the PHF6 fragment. (a) Side view of the molecule in the protein tunnel. (b) Diagonal view of the molecule in the protein tunnel. (PyMOL Software. The PyMOL Molecular Graphics System, Version 2.2.0 Schrödinger, LLC. URL: https://pymol.org/2/)

**Figure S5**


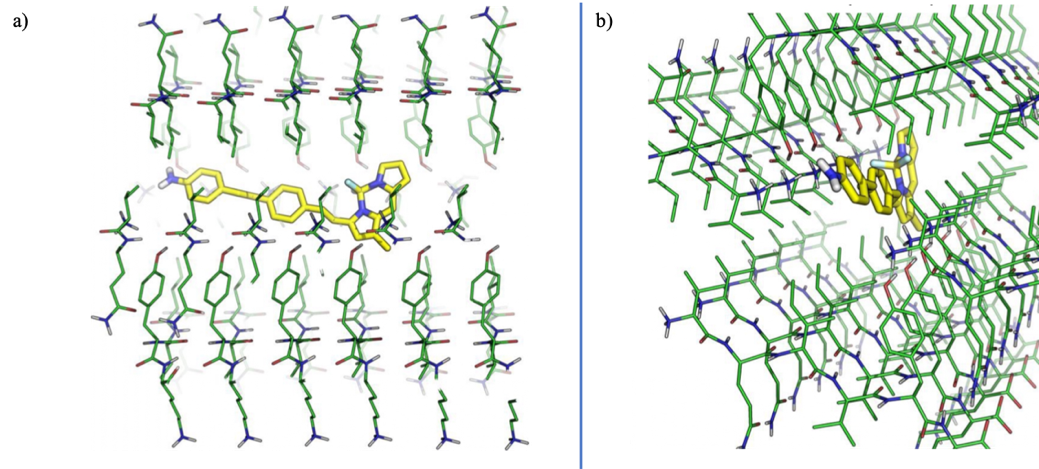


**Figure S5.** Molecular docking of BT4 against the crystallographic structure of the PHF6 fragment. (a) Side view of the molecule in the protein tunnel. (b) Diagonal view of the molecule in the protein tunnel. (PyMOL Software. The PyMOL Molecular Graphics System, Version 2.2.0 Schrödinger, LLC. URL: https://pymol.org/2/)

**Figure S6**


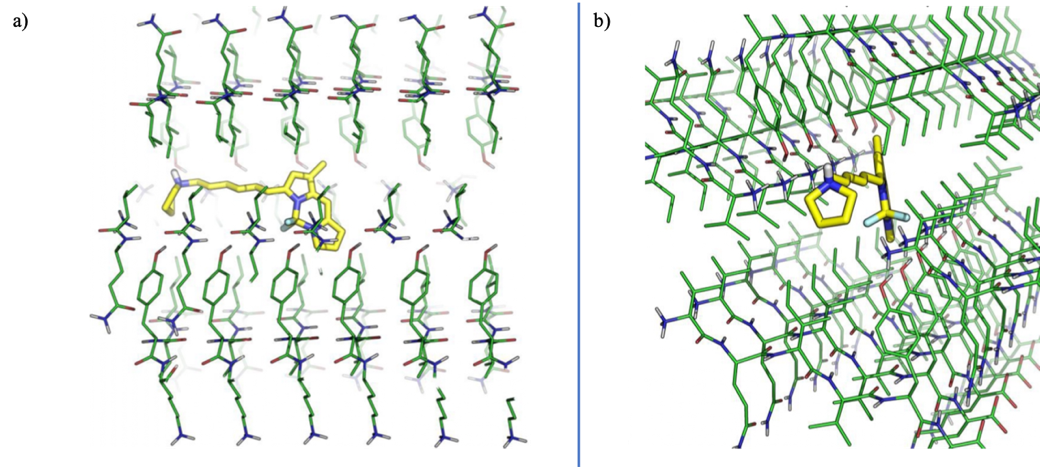


**Figure S6.** Molecular docking of BT5 against the crystallographic structure of the PHF6 fragment. (a) Side view of the molecule in the protein tunnel. (b) Diagonal view of the molecule in the protein tunnel. (PyMOL Software. The PyMOL Molecular Graphics System, Version 2.2.0 Schrödinger, LLC. URL: https://pymol.org/2/)

**Figure S7**


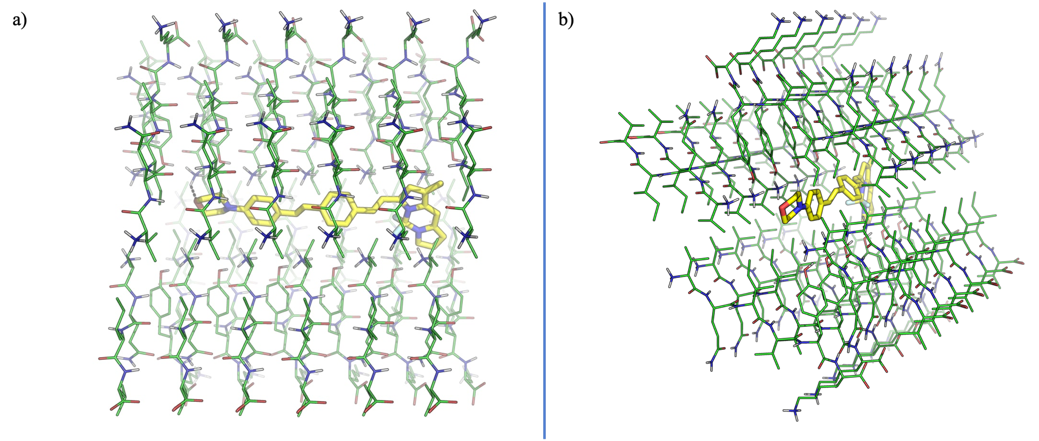


**Figure S7.** Molecular docking of BT6 against the crystallographic structure of the PHF6 fragment. (a) Side view of the molecule in the protein tunnel. (b) Diagonal view of the molecule in the protein tunnel. (PyMOL Software. The PyMOL Molecular Graphics System, Version 2.2.0 Schrödinger, LLC. URL: https://pymol.org/2/)

**Figure S8**


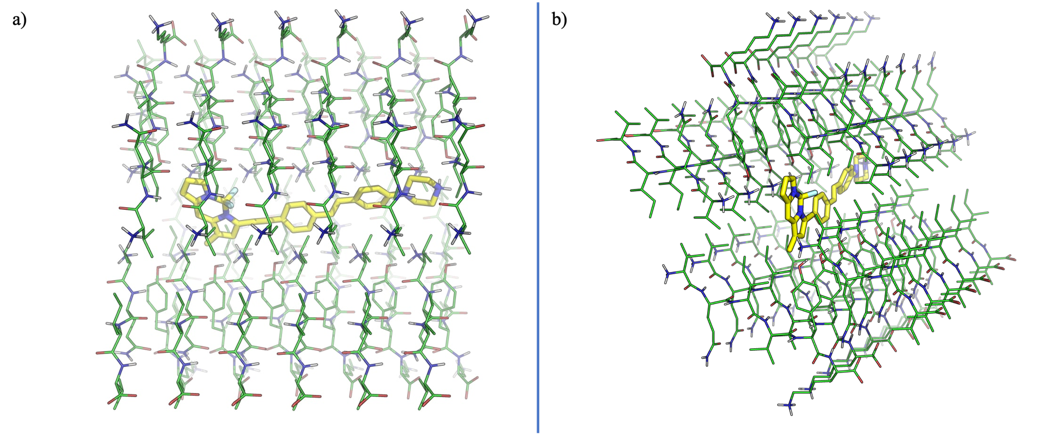


**Figure S8.** Molecular docking of BT7 against the crystallographic structure of the PHF6 fragment. (a) Side view of the molecule in the protein tunnel. (b) Diagonal view of the molecule in the protein tunnel. (PyMOL Software. The PyMOL Molecular Graphics System, Version 2.2.0 Schrödinger, LLC. URL: https://pymol.org/2/)

**Figure S9**


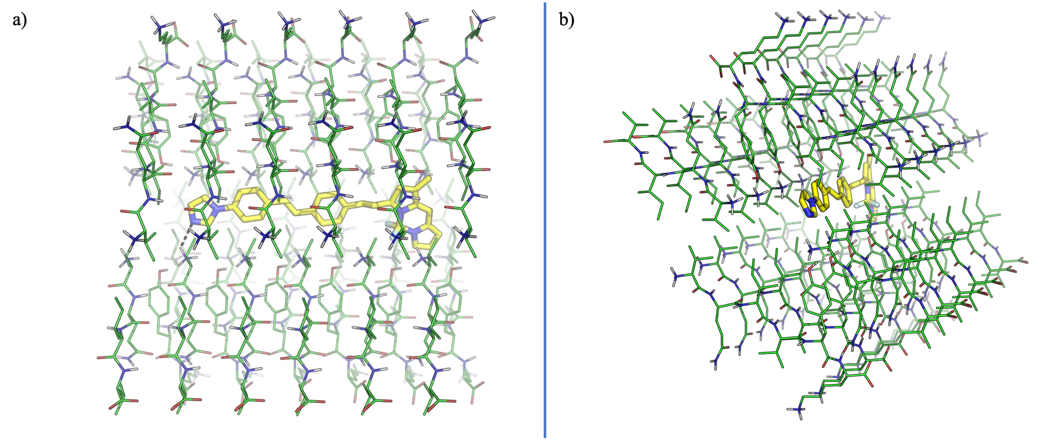


**Figure S9.** Molecular docking of neutral BT8 against the crystallographic structure of the PHF6 fragment: (a) Side view of the molecule in the protein tunnel. (b) Side view of the molecule protonated in the protein tunnel. (PyMOL Software. The PyMOL Molecular Graphics System, Version 2.2.0 Schrödinger, LLC. URL: https://pymol.org/2/)

**Figure S10**


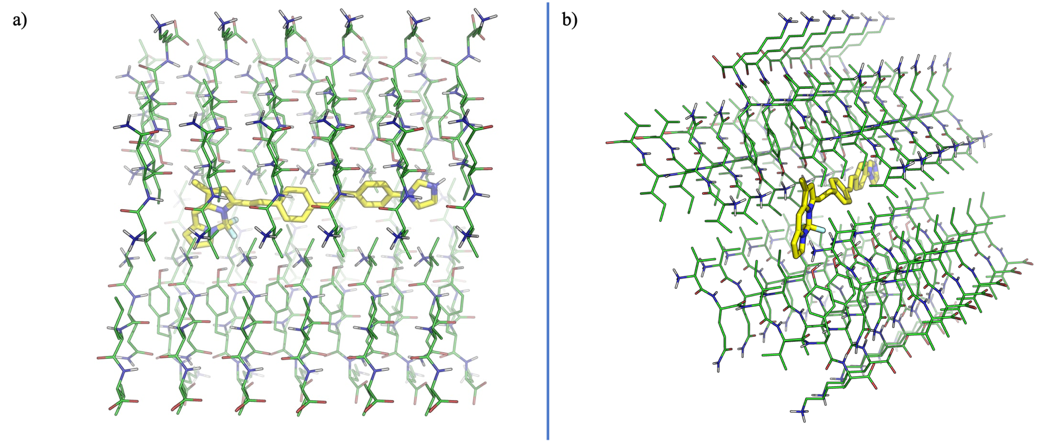


**Figure S10.** Molecular docking of protonated BT8 against the crystallographic structure of the PHF6 fragment: (a) Side view of the molecule in the protein tunnel. (b) Side view of the molecule protonated in the protein tunnel. (PyMOL Software. The PyMOL Molecular Graphics System, Version 2.2.0 Schrödinger, LLC. URL: https://pymol.org/2/)

**2. ^1^H and ^13^C NMR Spectra of compound 3**

**Figure S11**


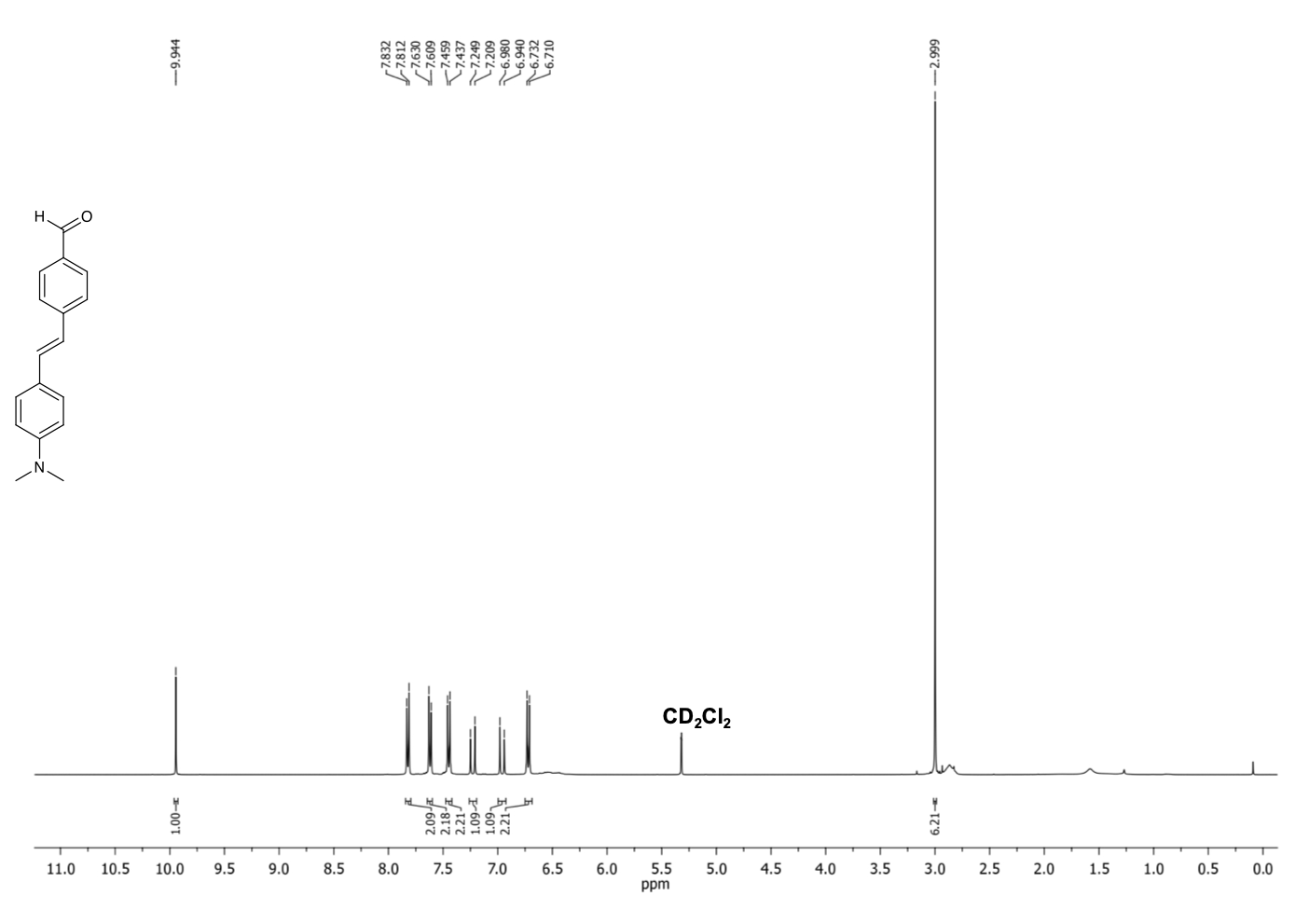


**Figure S11.** ^1^H NMR Spectrum (CD_2_Cl_2_, 400 MHz) of compound 3. (created by MestReNova version 12.0.0-20080)

**Figure S12**


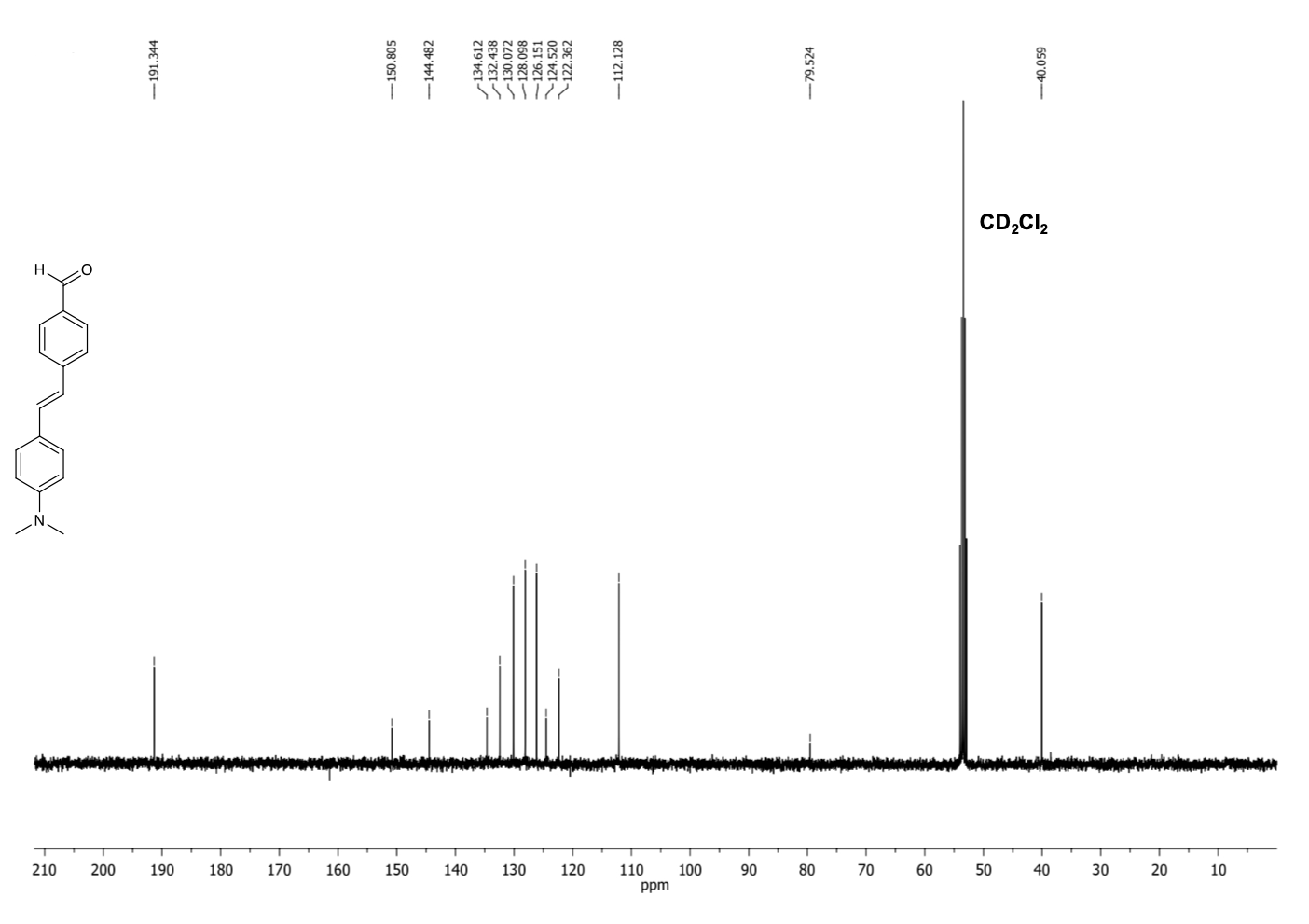


**Figure S12**. ^13^C NMR Spectrum (CD_2_Cl_2_, 100 MHz) of compound 3. (created by MestReNova version 12.0.0-20080)

**3. ^1^H and ^13^C NMR Spectra of BT1**

**Figure S13**


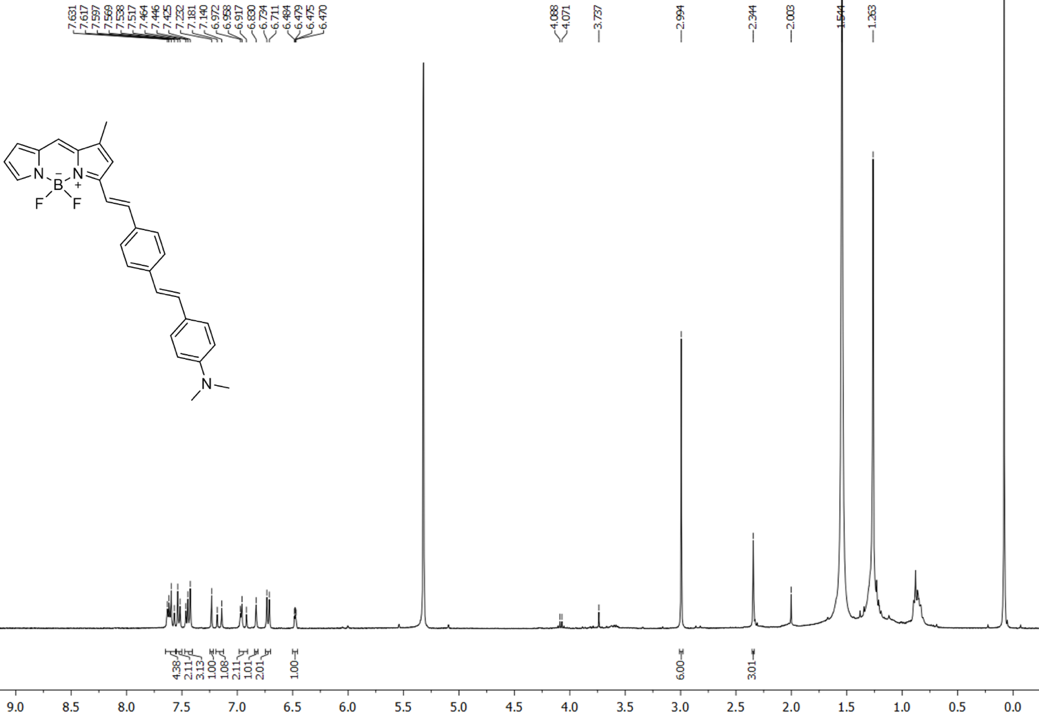


**Figure S13.** ^1^H NMR Spectrum (CD_2_Cl_2_, 400 MHz) of BT1. (MestReNova version 12.0.0-20080)

**Figure S14**


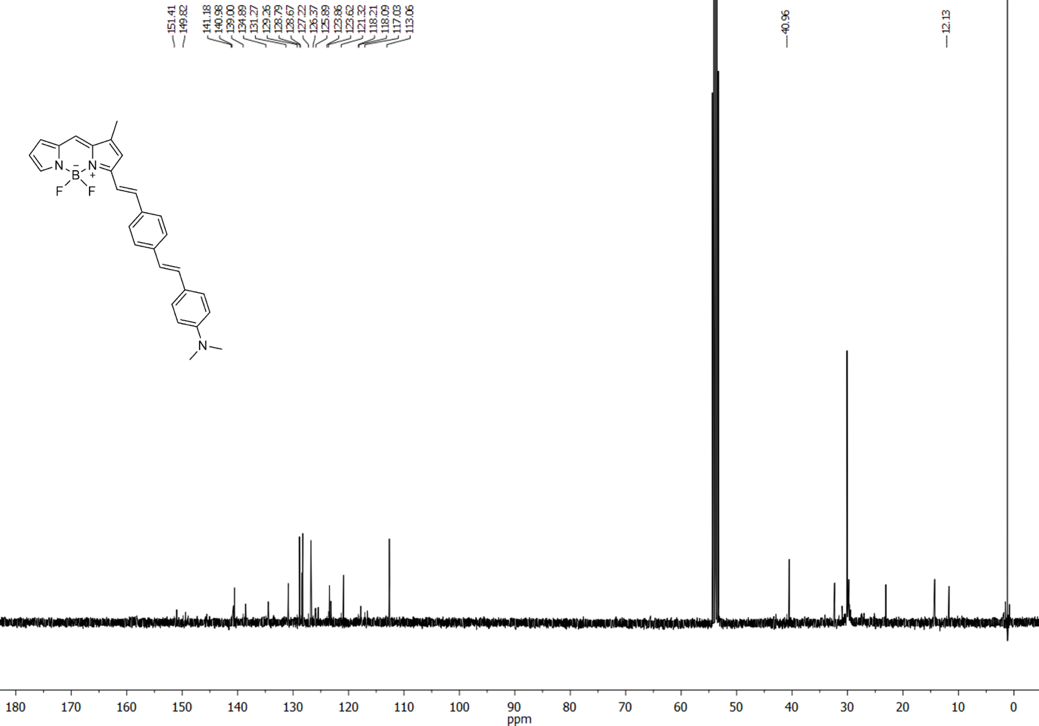


**Figure S14.** ^13^C NMR Spectrum (CD_2_Cl_2_, 100 MHz) of BT1. (MestReNova version 12.0.0-20080)

**4. ESI-MS Spectra of compound 3**

**Figure S15**


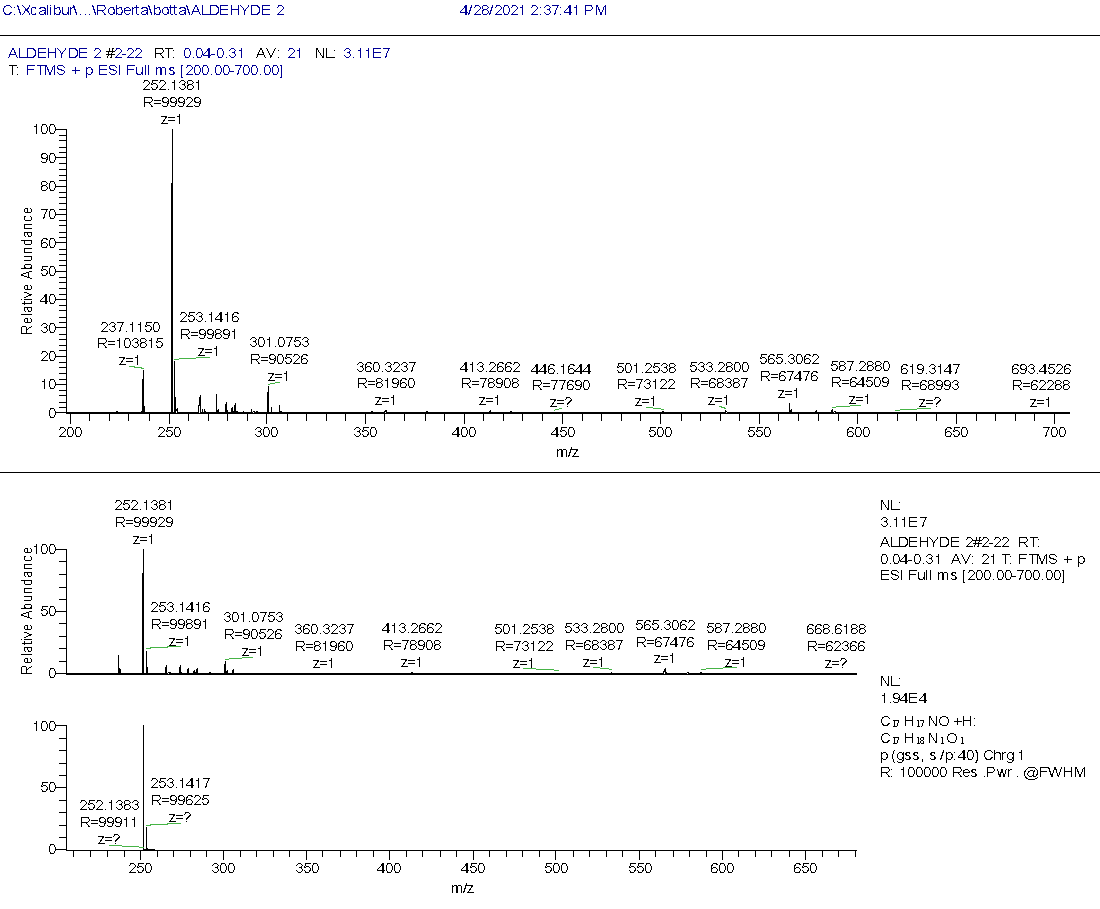


**Figure S15.** ESI-MS Spectrum of compound 3.

**5. ESI-MS Spectra of BT1**

**Figure S16**


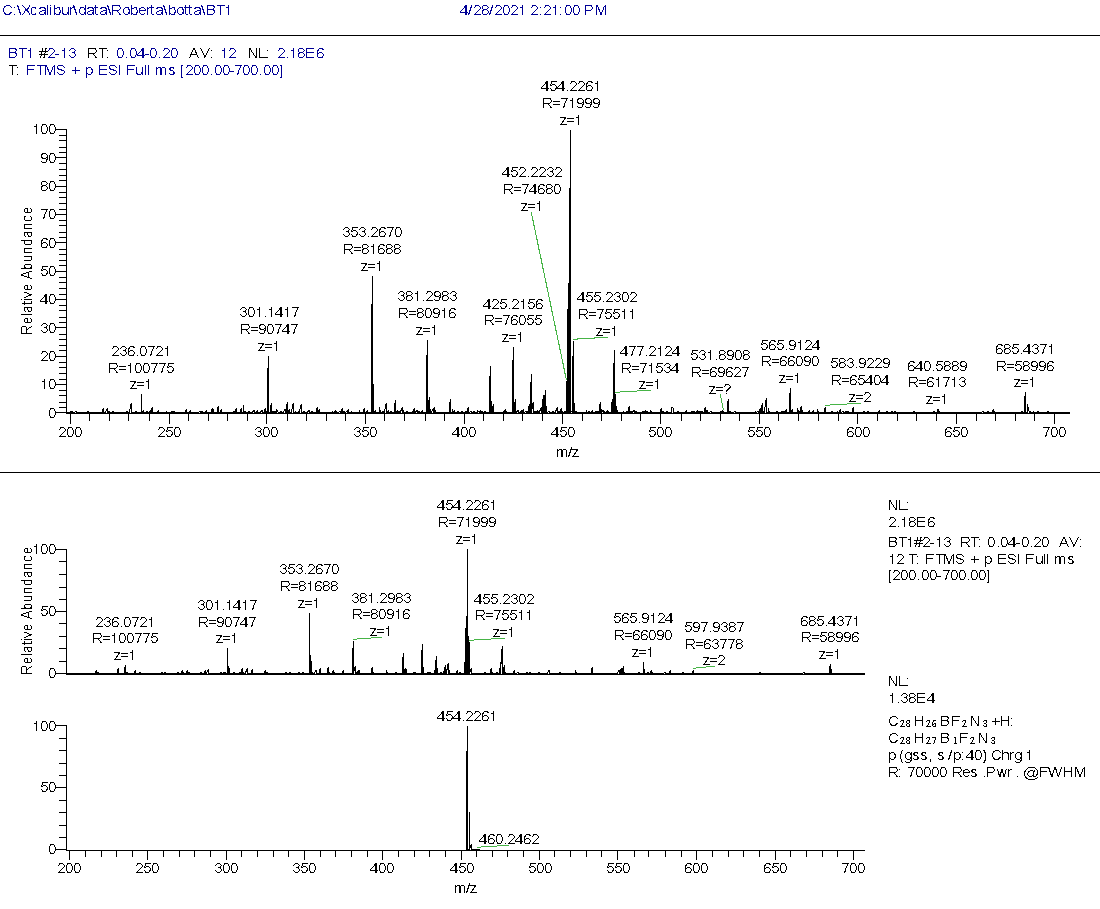


**Figure S16.** ESI-MS Spectrum of BT1.

**6. Fluorescence images of human iPSC WT#1 colony**

**Figure S17**


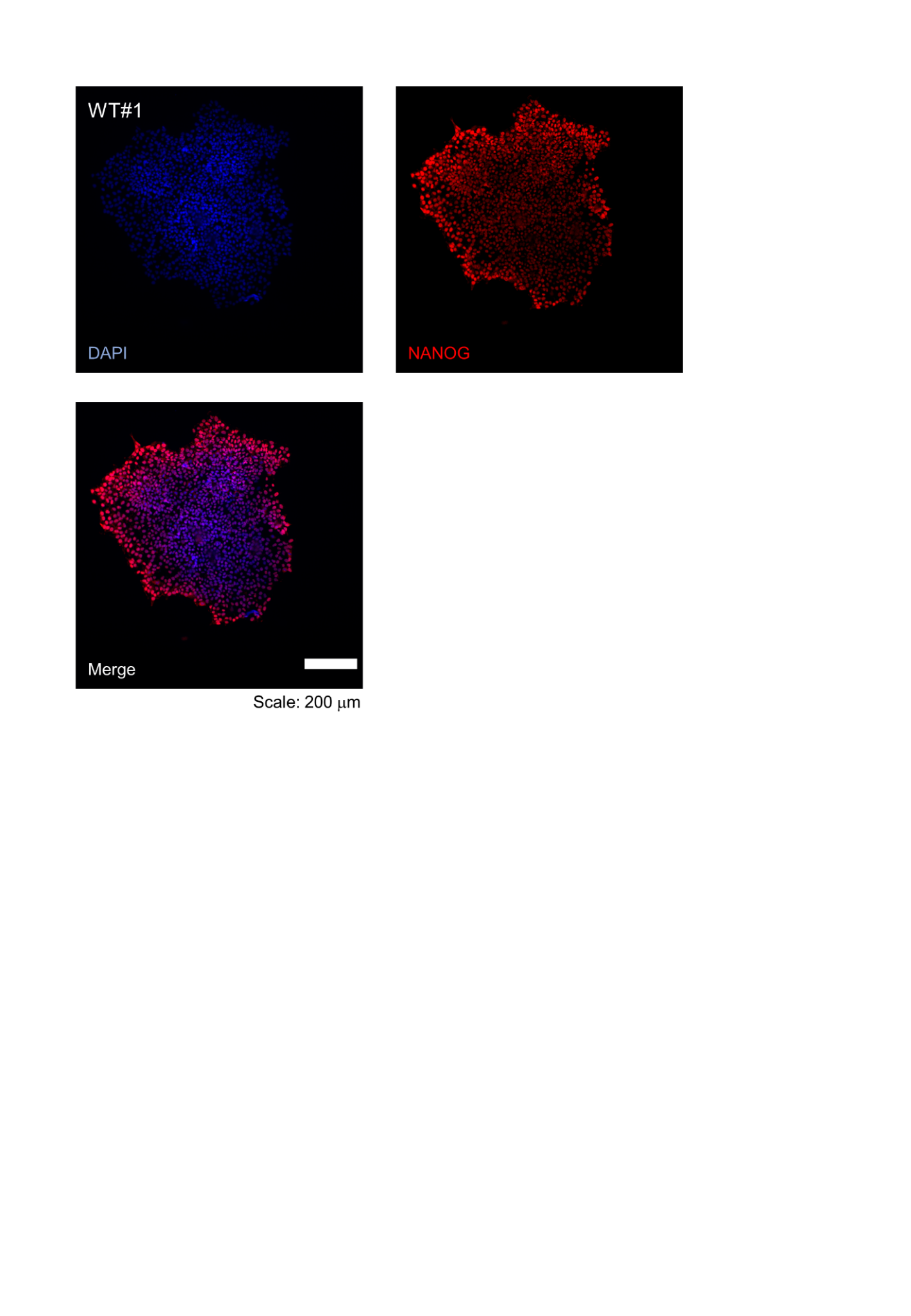


**Figure S17.** Representative fluorescence images of human iPSC colony stained for the pluripotency marker NANOG (red). Nuclei are stained with DAPI (blue) Scale bar: 200 μm. (ImageJ bundled with Java 1.8.0_172 software; URL: https://imagej.nih.gov/ij/)

**7. Emission spectra of TAU1**

TAU1 probe (purity >95%) fluorescence was analyzed when TAU1 was dissolved at 10 mM in DMSO and then diluted in PBS buffer (pH 7.4) to reach at a final concentration of 100 μM (1% DMSO) suitable for experiments with living neurons. Different excitation wavelengths, in the range 405- 640 nm, were selected and the corresponding emission spectra were recorded as shown in figure S18. Surprisingly, we found a very intense emission peak at 510 nm using as excitation wavelength 473 nm. As a possible explanation of this discrepancy with the previously reported spectra (ref Verwilst), in our experimental conditions TAU1 spectra were recorded in an aqueous solution and this may significantly affect the emission band if compared with an apolar solvent such as chloroform, which is not suitable for cell living (Hrdlovic et al., 2010).

**Figure S18**


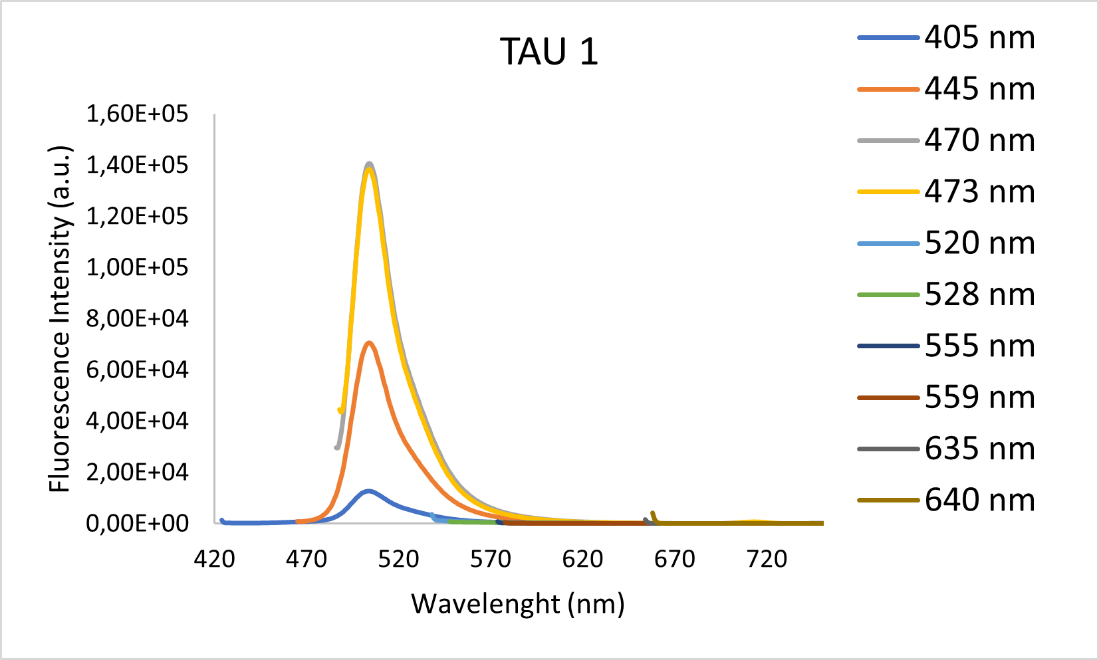


**Figure S18.** Emission spectra of purchased probe Tau 1 (100 μM) in PBS buffer pH 7.4 and 1 % DMSO according to the experimental conditions used for the immunostaining in human iPSC derived cortical neurons. The emission spectra were recorded at different λex as indicated in the legend on the right. (Ex. bandwidth = 10 nm; Em. bandwidth =10 nm). (GIMP software "The GIMP Development Team, 2019. GIMP, URL: https://www.gimp.org.)

Reference:

Hrdlovic P, Donovalova J, Stankovicova H, Gaplovsky A. Influence of polarity of solvents on the spectral properties of bichromophoric coumarins. Molecules. 2010;15(12):8915-8932

**8. Purification and fibrillogenesis assay of K18 domain of tau protein.**

8.1 Expression and purification of recombinant protein tau

Tau protein was designed from 244 to 376 amino acids and is referred to as the K18 domain which contains 4 microtubule-binding repeats (MTBR). K18 was expressed in E. coli BL-21 DE-3 strain and a high-density cell paste was purchased by Genscript Biotech Corporation®. Cell paste was homogenized and disrupted in a buffer containing (20 mM MES, 1 mM EDTA, 0,2 mM MgCl2, 300 mM NaCl, a tablet of cOmplete™ Protease Inhibitor Cocktail (Sigma-Aldrich), and 2 mM TCEP (Tris(2-carboxyethyl)phosphine hydrochloride)). The suspension was sonicated using a sonicator at 40% amplitude with pulse 4s on and 10s off for 15 min. The lysate was heated at 80°C for 20 min, and then centrifuged at 12000 rpm. Next, the clear supernatant was dialysed against 20 mM MES, 50 mM NaCl, 1 mM EDTA, 1mM MgCl2 and applied to a HiPrep SP 16/10 cation exchange column (Cytiva) connected to a ÄKTApure 25 (Cytiva) and pre-equilibrated with the same buffer. Then, the column was washed, and the protein was eluted with a step gradient of NaCl (0.2 M–0.8 M). tau was found to elute at 0.25 M NaCl. The purified fractions were pooled and dialyzed in a PBS buffer and then concentrated using 3 kDa cut off protein centrifugal filters. Separation of the monomeric from the higher oligomers was performed by Size exclusion chromatography applying protein samples in PBS on a column HiLoad® 26/600 Superdex® 75 (Cytiva) equilibrated in the same buffer. The calibration curve was calculated by measuring the retention time of conventional standards including human ferritin (MW = 480 kDa), bovine serum albumin (MW = 60 kDa), and cytochrome C (MW = 14 kDa). The concentration of tau was determined by UV absorption at 270 nm using an extinction coefficient of ε = 1490 M-1 cm-1 with a Jasco V-750 (Jasco Corporation, Tokyo, Japan). Purified protein was stored at –80°C in small aliquots

8.2 Transmission Electron Microscopy

The morphological characterization of tau fibrils has been carried out by bright-field transmission electron microscopy (TEM) using a CM100 electron microscope (Philips) equipped with high-contrast objective lens, a tungsten hexaboride (WB6) filament and a CMOS Phurona camera (Emsis). Micrographs have been acquired at 46000-130000X magnification while applying 100 keV electron energy on samples treated for negative staining as described herein. Two 20 μL drops of milliQ water and a 20 uL drop of 2% NanoW neutral stain solution (Ted Pella) have been placed on a piece of parafilm; 5 uL of sample solution have been placed onto the carbon surface of a 200 mesh carbon-coated copper grid (Ted Pella) gripped with the carbon-coated side facing up using negative locking tweezers and let to adsorb 30 s before blotting off the sample volume by touching the grid’s edge with filter paper (Whatman); the sample has been then washed by touching the carbon-coated side with the water drop followed by blotting off with filter paper; for staining, the sample has been touched with the stain drop for 1 min before blotting off the stain solution with filter paper; a second washing step has carried out as described above and the grid has been air dried 20 min with the carbon-coated side facing up before TEM imaging.

**Figure S19**


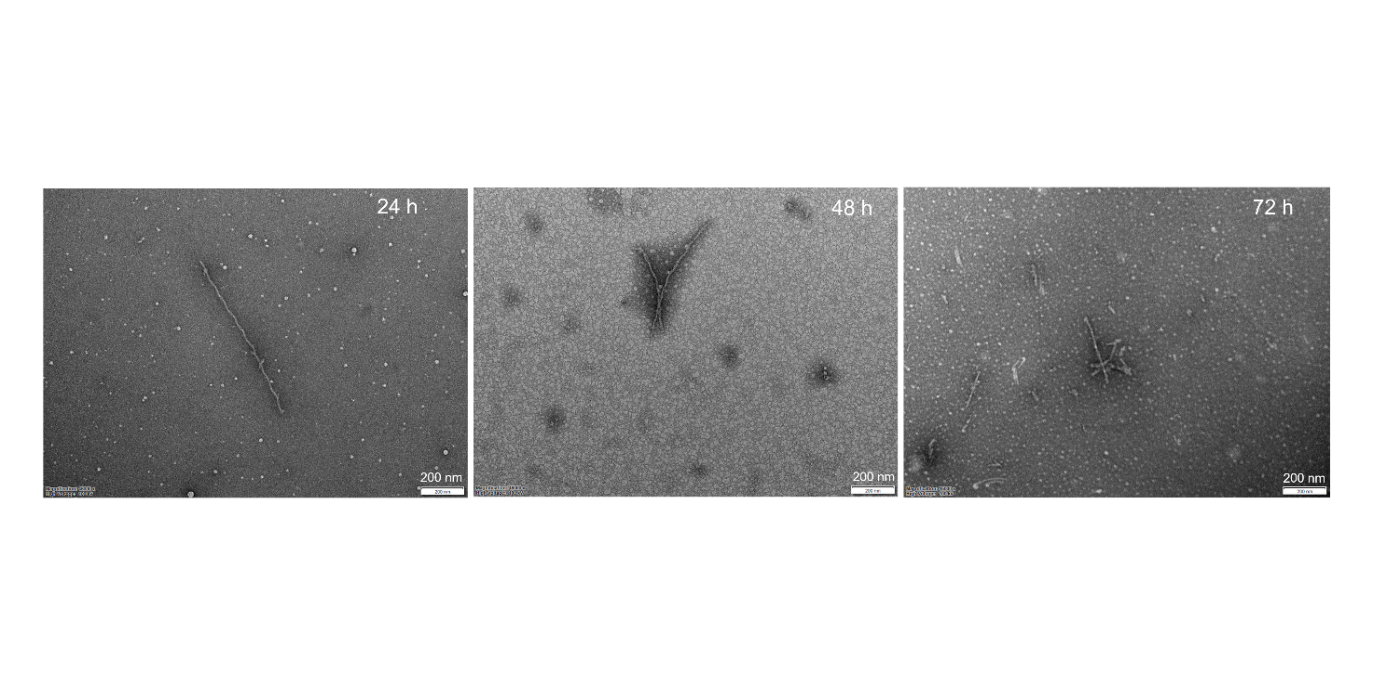


**Figure S19.** TEM images of tau K18 protein incubated with heparin (ratio 1:1) at 37°C after 24 h, 48 h, 72 h confirming the formation of regular fibrils of tau.

**9. Reagents**

**Table S2**


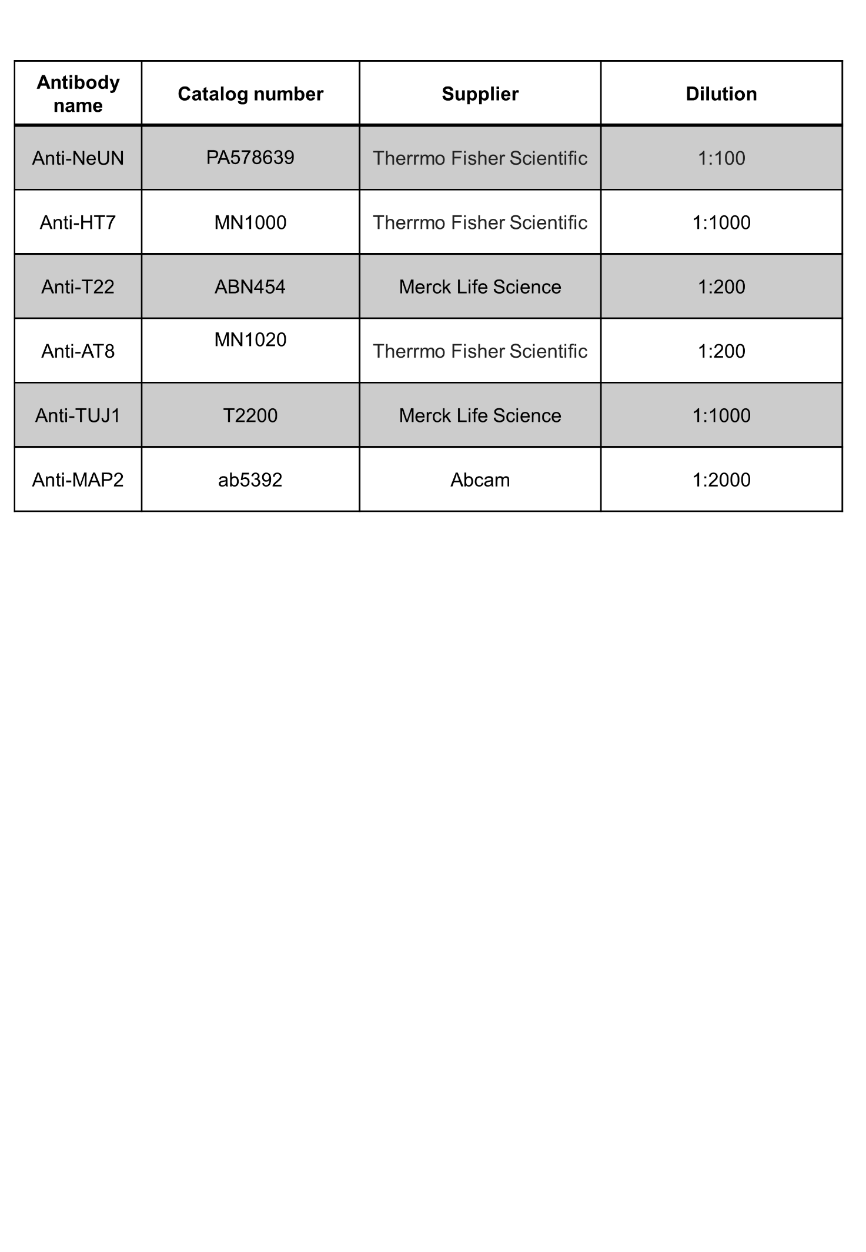


**Table S2.** List of antibodies.

**Table S3**


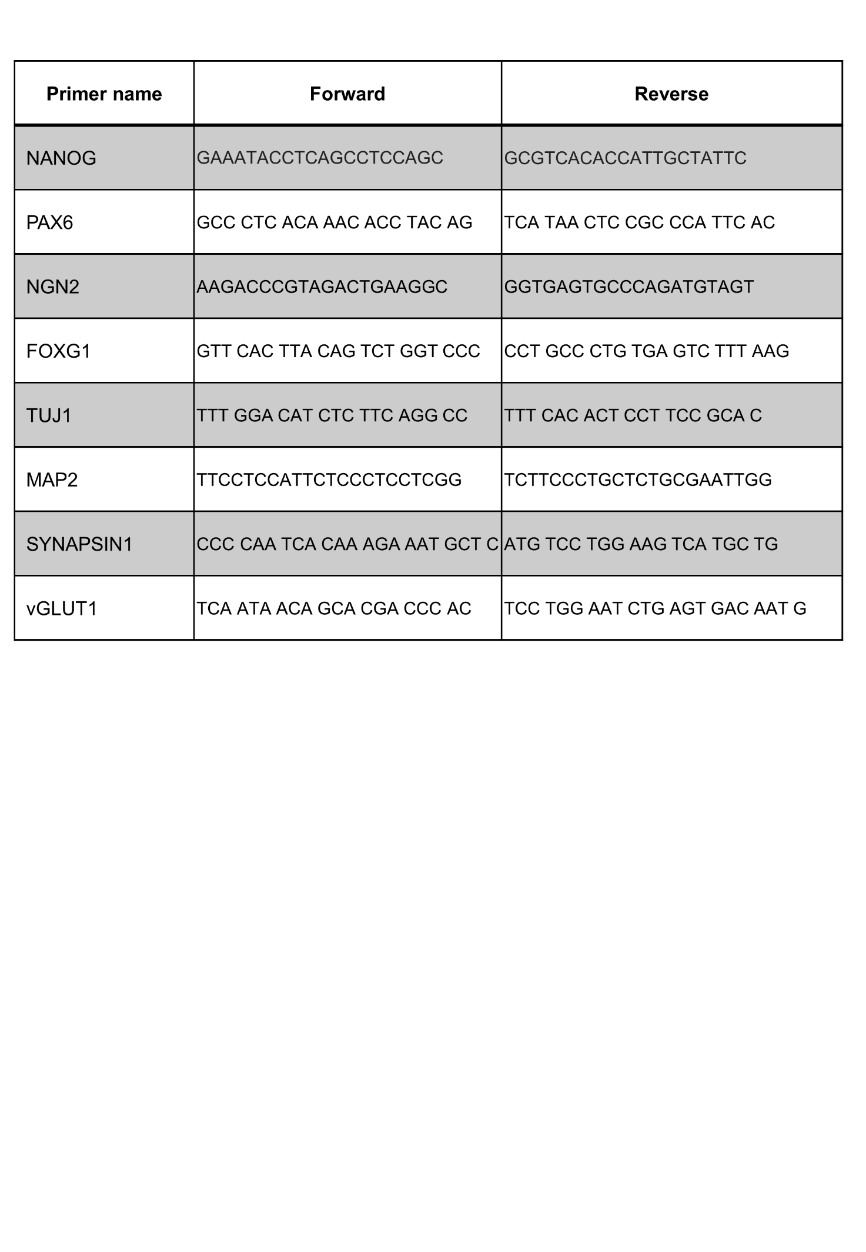


**Table S3.** List of primers used for evaluating the iPSC differentiation.
